# General discussion of data quality challenges in social media metrics: Extensive comparison of four major altmetric data aggregators

**DOI:** 10.1371/journal.pone.0197326

**Published:** 2018-05-17

**Authors:** Zohreh Zahedi, Rodrigo Costas

**Affiliations:** 1 Centre for Science & Technology Studies (CWTS), Leiden University, Leiden, the Netherlands; 2 DST-NRF Centre of Excellence in Scientometrics and Science, Technology and Innovation Policy, Stellenbosch University, Stellenbosch, South Africa; Indiana University Bloomington, UNITED STATES

## Abstract

The data collection and reporting approaches of four major altmetric data aggregators are studied. The main aim of this study is to understand how differences in social media tracking and data collection methodologies can have effects on the analytical use of altmetric data. For this purpose, discrepancies in the metrics across aggregators have been studied in order to understand how the methodological choices adopted by these aggregators can explain the discrepancies found. Our results show that different forms of accessing the data from diverse social media platforms, together with different approaches of collecting, processing, summarizing, and updating social media metrics cause substantial differences in the data and metrics offered by these aggregators. These results highlight the importance that methodological choices in the tracking, collecting, and reporting of altmetric data can have in the analytical value of the data. Some recommendations for altmetric users and data aggregators are proposed and discussed.

## Introduction

*Altmetrics* offer the possibility of studying new forms of interactions between social media users, scholarly objects, and different academic actors. As such, altmetrics or rather social media metrics [1] have paved the way towards the study of the relationships and interactions between social media and scholarly entities in what can be seen as the *social media studies of science* [2]. However, for the proper development of this new genre of studies it is critical to understand all possible data quality challenges in the capture of social media events around scholarly objects [3]. Therefore, questions such as *from where*, *when* and *how* social media data has been collected and processed become critical in the development of reliable and replicable social media metrics research. Besides, the existence of different altmetric data aggregators opens the question of how these aggregators are approaching the collection of social media data; and how their different approaches may introduce discrepancies in the results based on their data. The study of social media metrics data quality is a critical and central element in the further development of altmetric research.

### Data issues in bibliometrics

The importance of relying on reliable and valid data has always been a bone of contention in bibliometric research. The occasional lack of transparency of some bibliometric databases (e.g. Web of Science, Scopus, Google Scholar), together with the errors and inconsistences found in citations, have been often highlighted in the literature [4; 5; 6], particularly regarding their potential effect in research evaluation. For instance, errors such as inaccurate cited references and duplicate records in Scopus or Web of Science [7] have been discussed to have serious consequences in the calculation of citation indicators for journals, individuals, or institutions [8; 9]. Given the importance of the data quality of bibliometric data, comparative analyses of bibliometric and citation data sources have proliferated [10], often providing recommendations on how to improve the accuracy and quality of bibliometric databases [4].

### Data issues in social media metrics data

In the case of social media metrics data sources much less is known about their potential issues regarding their data quality. Different approaches in collecting, processing, reporting, and updating the data have been discussed to largely influence the social media metrics offered by different altmetric aggregators [11; 12]. The possibility to track the provenance of the original data is considered an important aspect regarding the verification of the data and metrics provided [13]. However, only few studies have systematically compared different altmetric aggregators based on their coverage of publications and calculation of metrics [13; 14; 15; 16; 17]. These previous studies have pointed out that the social media metrics data reported can be influenced by issues related with the different timing of data collection, different sources (of for example blog lists or main stream news) tracked, use of APIs (commercial vs. public), or the choice of publication identifiers (e.g. DOIs, PMIDs) to access and track social media data. Therefore, similar to citation data [18;19;20], it is important to understand how variations in the social media metrics reported by the different aggregators may influence the results obtained.

A more recent study [21] showed that major altmetric aggregators (Plum Analytics, Altmetric.com, CrossRef Event Data) provide different metrics for the same set of papers. These differences challenge the reliability of social media metrics. Possible solutions could be just to select some specific sources (e.g. those that provide the highest scores or coverage) or even their combination, as it seems to be suggested by Ortega (2017) [21]. However, for example the selection of aggregators with higher scores do not necessarily mean better indicators or data. For example, higher scores can be caused by the combination of different recorded actions coming from the same social media source (e.g. by counting under the same indicator Facebook shares, likes and wall posts publications, instead of keeping them as separate metrics) or by aggregating metrics from duplicate records of the same object. Such choices may cause even more unreliable results, since different sources of error could be merged in the same indicator. Hence, from our point of view, it is very important to understand the underlying reasons of the existing differences. Also, it is important to discuss how different methodological and technical choices can influence the metrics provided. From this perspective, we aim at providing a more reasoned discussion of the current challenges of social media metrics data, instead of a mere recollection of who is providing the higher (lower) scores or the description of data issues in altmetric sources.

### Altmetric data aggregators

Among the most important altmetric aggregators currently collecting and providing social media metrics we can highlight Altmetric.com, Lagotto, Plum Analytics, and CrossRef Event Data. These altmetric data aggregators offer access to data and metrics related with the online activity and social media interactions between social media users and scholarly objects. We also include here the description of social media metrics (number of readers) obtained from Mendeley.com. Although Mendeley.com is an altmetric data provider and not an altmetric data aggregator [22], it is included in this study in order to compare the results of a direct data collection from Mendeley with that of other aggregators. Thus it is possible to better discuss the potential differences found in Mendeley metrics provided by the different aggregators with a common benchmark (i.e. our own data collection from Mendeley). In order to simplify the terminology throughout the paper we will refer to all of them as aggregators, even if we sometimes refer to Mendeley.com.

#### Altmetric.com (http://www.altmetric.com/)

*Altmetric*.*com* is a Digital Science company founded in 2011 and based in London (United Kingdom). More than 64 million mentions of 9 million research outputs are covered by Altmetric.com database in January 2018 (https://www.altmetric.com/about-our-data/how-it-works/). A range of different sources (https://www.altmetric.com/about-our-data/our-sources/) including mentions in policy documents, blogs, mainstream media, online reference managers, and social media tools, etc. are tracked for URLs or scholarly outputs unique identifiers (e.g. PubMed ids, ArXiv ids). Counts for each tracked object (journal articles, datasets, images, reports,) are available via its detail page (https://www.altmetric.com/details/950642) and the recorded data are available for free for researchers through the Altmetric API with a rate limit.

#### CrossRef Event Data (www.eventdata.crossref.org/)

*Crossref Event Data* (CrossRef ED) is a service started in April 2017 and is in its beta version. Event Data (https://www.eventdata.crossref.org/guide/data/about-the-data/-sources-and-agents) collects raw data from a selection of sources (https://www.eventdata.crossref.org/guide/data/about-the-data/) such as Wikipedia, Twitter, Reddit, Stack Exchange Network, etc. for CrossRef registered contents. This service connects to some external data sources via its agent for turning the data into ‘events’ (bookmarks, comments, shares) and provides provenance, context, and links for each event. The resulted events are publically available via an open Event Data API. It is important to highlight that this service doesn’t provide metrics but a stream of Events (raw data) that occurred for a given piece of registered content with a DOI [23].

#### Lagotto open source application (www.lagotto.io/)

*Lagotto* is an Open Source application started in March 2009 by the Open Access publisher Public Library of Science (PLOS). Lagotto started by providing social media mentions of PloS articles and later also for articles from any other publisher. Lagotto retrieves (version 4.2.1. released on 13 July 2015) data from a wide set of services and sources (http://www.lagotto.io/docs/sources/). The metrics are grouped in different categories of impact (viewed, saved, cited, and recommended) and are available through an open API (http://www.lagotto.io/docs/api/).

#### Plum Analytics (https://plumanalytics.com/)

*Plum Analytics* was founded in 2012, acquired by Ebsco in 2014 and by Elsevier in 2017. *Plum Analytics* provides metrics for different research outputs (articles, blog posts, books, source codes, theses/dissertations, videos) via its ‘artifact’ [object] level page. The metrics are grouped in 5 categories of usage, captures, mentions, social media, and citations. PlumX is a subscription-based platform and hence no open API is available; however, artifact-level PlumX pages are free and publicly accessible.

#### Mendeley (http://www.mendeley.com/)

*Mendeley* is a free online reference manager and academic social network founded in 2007 and acquired by Elsevier in 2013. This platform is used by over 6 million users worldwide. It offers ‘readership’ statistics capturing the number of different Mendeley users that have saved a given publication (together with their academic statuses, countries and disciplines).

### Methods of collecting, tracking, and updating social media metrics

Although these aggregators may collect data from similar data sources (e.g. Twitter, Facebook, Wikipedia, Mendeley), it is common that they adopt different methodological approaches when collecting, processing, and reporting the data [16]. From a conceptual point of view, we argue that there are three main central elements in the systematization of the different methodological approaches adopted by the different altmetric data aggregators:

**Data collection approaches**. Not all altmetric aggregators track the same document types (books, reviews, articles, datasets, slides), journals, or publishers. They also vary in the social media sources they cover (e.g. Facebook, Twitter, Wikipedia, Mendeley). Aggregators may also use different APIs to access the primary sources (e.g. Altmetric.com, Plum Analytics, and CrossRef ED use GNIP for Twitter data while Lagotto uses Search API).
**Aggregation and reporting approaches**. Aggregators may differ in recording public vs. private data (e.g. public walls Facebook posts are tracked by Altmetric.com while Lagotto and Plum Analytics also track private posts and shares). Social media metrics may be reported with different degrees of detail, thus moving from mere counts or summaries of events to providing the raw metadata collected (e.g. CrossRef ED provides the raw data collected and no counts, while aggregated data at the output level are displayed in Plum Analytics, Altmetric.com, or Lagotto). Aggregators also differ in the scholarly object identifiers (DOIs, PMIDs, Arxiv IDs) they track. Different data processing approaches may also be used. Some aggregators may choose to aggregate tweets and retweets in one single count (e.g. Lagotto), keep them separate (e.g. Plum Analytics, although in their total count they sum them together), just provide the count of the distinct tweeters around a publication (e.g. Altmetric.com), or the raw metadata of the (re)tweets mentioning the scholarly objects (e.g. CrossRef ED).
**Updating approaches**. Different criteria to update the social media data (daily, weekly) are also applied by the different altmetric aggregators.

## Aim of the study

As explained above, there are indeed similarities and differences on how data aggregators approach the data collection, processing, and update of social media events around different scholarly objects and their identifiers. Given these disparities, there is a critical need of understanding how these differences can cause variations in the nature and characteristics of the social media metrics provided. This understanding is fundamental for the future development of robust and reliable applications, ensuring “transparency”, “accuracy”, and “replicability” as suggested in the literature [22; 24]. Hence, we aim not just at *identifying the potential discrepancies* but also at *conceptualizing the reasons and implications that these differences may have for further social media metrics research*. The paper is organized as follows. In section 2 the main methodological design of this study is described. Section 3 is structured as follows, a first part (section 3.1) includes a quantitative analytical description of the data discrepancies across aggregators. In a second part (section 3.2), a general discussion of potential reasons for the differences found is presented. Finally, some general conclusions and recommendations for social media metrics researchers and altmetric data aggregators are introduced in section 4.

## Data and methodology

Publications with a DOI published in PloS ONE (n = 31,437) in 2014 and available in the CWTS in-house version of Web of Science (WoS) database have been considered in this study. PloS ONE publications were chosen since they are covered and tracked by all aggregators considered for this study. The DOIs of these publications were used to collect social media metrics data from the described altmetric aggregators using their APIs or dedicated websites:

- Altmetric.com REST API (http://api.altmetric.com/);
- CrossRef Event Data API (www.eventdata.crossref.org/guide/service/query-api/);
- Lagotto open source application API (www.lagotto.io/docs/api/);
- Plum Analytics (https://plu.mx/plum/a/?doi=[doi]);
- Mendeley REST API (http://dev.mendeley.com/).

The data collection from all the selected altmetric data aggregators was done in exactly the same date: *2017 June 19*^*th*^ with the aim of minimizing time effects in the data collection. Altmetric data from *Facebook*, *Twitter*, *Mendeley*, and *Wikipedia* obtained from these aggregators were considered for comparisons. Some descriptive statistics such as the sum and average scores of different metrics, and the coverage of publications (% of publications captured by each altmetric aggregators with at least one metric in each of the tracked sources) have been calculated. The (dis)agreement of the metrics provided among altmetric aggregators have also been studied, and Pearson correlations have been calculated in order to determine the relationship between the metrics provided by the aggregators. Possible reasons for the differences found are summarized and discussed, particularly regarding the further development of research and applications of social media metrics.

## Results

In this section, first, we present the differences across aggregators including discrepancies in the coverage of publications, total counts of all and overlapped publications, and correlation analysis of metrics across aggregators.

### Differences across aggregators

#### Coverage of publications

The main results of the coverage of publications with some social media recorded activity are presented in Table 1. Overall, Plum Analytics has the highest coverage (99.9%) of PloS ONE publications, followed by Lagotto (99.8%), Mendeley.com (95.9%), and Altmetric.com (61%). CrossRef ED has the lowest coverage (7.5%) of all PloS ONE publications considered in this study. The coverage of publications per data source is presented in the following columns.

**Table 1:** Coverage (% of DOIs with at least one metric) of PloS ONE DOIs across altmetric aggregators and aggregators and per data sources.

**Aggregators**	**No. publications with coverage (% pubs)**	**No. publications on Mendeley (% pubs. On Mendeley)**	**No. publications on Twitter (% pubs. On Twitter)**	**No. publications on Facebook (% pubs. On Facebook)**	**No. publications on Wikipedia (% pubs. On Wikipedia)**
**Altmetric.com**	19,185	19,073	17,926	3,623	639
	*(61*.*0)*	*(60*.*6)*	*(57*.*0)*	*(11*.*05)*	*(2*.*0)*
**CrossRef ED**	2364	N/A	555	N/A	716
	*(7*.*5)*	*N/A*	*(1*.*7)*	*N/A*	*(2*.*2)*
**Lagotto**	31,398	30,117	9,973	2,497	1,615
	*(99*.*8)*	*(95*.*8)*	*(31*.*7)*	*(7*.*9)*	*(5*.*1)*
**Mendeley.com**	30,154	30,124	N/A	N/A	N/A
	(95.9)	(95.8)	*N/A*	*N/A*	*N/A*
**Plum Analytics**	31,418	30,389	7,526	5,149	747
	***(99*.*9)***	***(96*.*6)***	***(23*.*9)***	***(16*.*3)***	***(2*.*3)***

(N/A: metrics not available in the platform)

**Mendeley coverage**. Regarding the coverage of publications with at least one Mendeley reader, Plum Analytics has the highest coverage of Mendeley readerships (96.6%), even higher than Mendeley itself (95.8%), followed by Lagotto (95.8%), and Altmetric.com (60.6%). The substantial lower coverage of Altmetric.com is caused by the data collection policy of this aggregator (see 3.2.1 section). CrossRef ED does not collect Mendeley readership.

**Twitter coverage**. Altmetric.com exhibits the largest coverage of publications with at least one tweet (57%), followed by Lagotto (31.7%), Plum Analytics (23.9%), and CrossRef ED (1.7%). This lower coverage of tweets by CrossRef ED can be related to the recent start of this service, which is still in its Beta version (www.eventdata.crossref.org/guide/index.html).

**Facebook coverage**. Regarding publications with some coverage on Facebook, Plum Analytics (16.3%) has the largest coverage followed by Altmetric.com (11.5%), and Lagotto (7.9%) while Crossref ED does not collect any Facebook mentions at this moment.

**Wikipedia coverage**. Lagotto (5.1%) has the highest share of publications with at least one mention in Wikipedia, followed by Plum Analytics (2.3%), CrossRef ED (2.2%), and Altmetric.com (2%).

#### Total counts

In Table 2 the total sum of counts for all publications in each of the studied indicators is presented. Mendeley readership counts include the sum of all readership counts reported by each of the altmetric aggregators. For Altmetric.com the total number of (re)tweets per publication recorded by this source has been calculated by ourselves. The aggregator does not provide per se the total number of (re)tweets a publication has received but the distinct number of tweeters that have (re)tweeted the publication. For CrossRef ED, the count of (re)tweets, number of distinct (re)tweeters, and the count of Wikipedia mentions are calculated by ourselves based on the raw data provided in their JSON files. Plum Analytics already reports the sum of all (re)tweets, Facebook, and Wikipedia counts. It also provides a breakdown by tweets and retweets. The total counts of (re)tweets provided by Plum Analytics is used in this study.

**Table 2:** Statistics (sum [t] and mean [m] scores) of altmetric counts across aggregators and per data source.

**Aggregators** **nP = 31,437**	**tMR**	**mMR**	**tTW**	**mTW**	**tFB**	**mFB**	**tW**	**mW**
**Altmetric.com** **CrossRef ED** **Lagotto** **Mendeley.com** **Plum Analytics**	491,630 N/A 679,898 653,283 671,834	15.6 N/A 21.6 20.8 21.4	164,919 (143,471) 2,912 (2,359) 104,840 N/A 76,113	5.2 (4.5) 0.1 (0.07) 3.3 N/A 2.4	22,627 N/A 67,073 N/A 275,122	0.7 N/A 2.1 N/A 8.8	1,060 11,221 4,683 N/A 1,135	0.0 0.4 0.1 N/A 0.0

nP = number of Publication; t = sum score; m = mean score; MR = Mendeley readership counts, TW = (re)tweets, FB = Facebook counts, W = Wikipedia mentions, N/A = metrics not available in the platform, values in parentheses refer to statistics of distinct tweeters (Twitter users)—only for Altmetric.com and CrossRef ED.

According to Table 2, Lagotto and Plum Analytics provide the highest counts of Mendeley readership (tMR) outperforming the counts provided by Mendeley itself. In terms of Twitter counts (tTW), Altmetric.com reports the highest counts of tweets while CrossRef ED presents the lowest values. Plum Analytics provides the highest value of Facebook counts (tFB) and CrossRef ED provides the highest value of Wikipedia counts (tW).

#### Counts of overlapped publications

Tables 1 and 2 show that there are indeed differences in the coverage and counts provided by the aggregators. Thus, it is important to delve into the main possible reasons behind these differences. In order to do so, we first explore the level of (dis)agreement in the values of those publications that are covered by the same pairs of altmetric aggregators (i.e. overlapped publications between aggregators).

**Mendeley readership counts**. A total of 19,073 publications (60.6% of the total) are covered by both Altmetric.com and Mendeley. Of these, 18,613 publications (97.9%) have exactly the same number of readership counts (Table 3). This suggests a strong agreement between Altmetric.com Mendeley data and our method to extract data from Mendeley (as described in Section 2). A total of 153 publications (0.8%) have higher scores recorded in Altmetric.com, while 249 publications (1.3%) have lower scores in Altmetric.com than in Mendeley.

**Table 3:** Analysis of (dis)agreement among aggregators in Mendeley readership counts.

	**Mendeley.com** **(n = 30,124)**				**Altmetric.com** **(n = 19,073)**				**Lagotto** **(n = 30,117)**			
readerships	overlapped	equal	>	<	overlapped	equal	>	<	overlapped	equal	>	<
**Altmetric.Com** **(n = 19,073)**	19,015	18,613	153	249								
**%**		97.9%	0.8%	1.3%								
**Lagotto** **(n = 30,117)**	30,117	14,416	13,974	1,727	19,012	7,977	9,823	1,212				
**%**		47.9%	46.4%	5.7%		42.0%	51.7%	6.4%				
**Plum Analytics** **(n = 30,389)**	30,089	9,027	10,531	10,531	19,057	5,120	6,974	6,963	30,086	7,676	7,815	14,595
**%**		30.0%	35.0%	35.0%		26.9%	36.6%	36.5%		25.5%	26.0%	48.5%

In the case of Lagotto, a total of 30,117 publications (95.8%) are covered in both Lagotto and Mendeley, of which 14,416 publications (47.9%) have exactly the same readership score as reported by Mendeley. In contrast, 13,974 publications (46.4%) have higher scores in Lagotto and 1,727 publications (5.7%) have lower scores in Lagotto than in Mendeley. This suggests a relatively weaker agreement between our method to query Mendeley and that of Lagotto.

For Plum Analytics, although it exhibits the largest coverage of publications with Mendeley readership counts (30,089 publications, 96.6%), only 30% of publications have exactly the same scores as reported by Mendeley (as based on our DOI-approach for querying the API). Hence, there is a strong disagreement in the readership scores (a total of 70% of publications with higher or lower scores) reported by Plum analytics and our Mendeley data collection approach.

When we compare the agreements between the rest of pairs of altmetric aggregators, Altmetric.com and Lagotto exhibit the strongest agreement in their Mendeley scores (42%), while Altmetric.com and Plum Analytics have a much lower agreement (26.9%). An even lower agreement (only 25.5% of the 30,086 overlapped publications) is found between Lagotto and Plum Analytics. An explanation for these discrepancies could be the fact that Plum Analytics merges the Mendeley counts for different identifiers across different versions of the same publication, while our approach was partly limited by the DOI-querying of the Mendeley API, which seems to be used by other aggregators, particularly Altmetric.com and Lagotto (see section 3.2.1).

**Tweets and tweeters counts**. When it comes to the analysis of Twitter data, Altmetric.com presents the highest number of (re)tweets as compared to other aggregators. Overall, we notice a lower agreement in the Twitter scores reported by all aggregators (Table 4) as compared to Mendeley. The largest set of overlapped publications is found between Altmetric.com and Lagotto, with 9,763 publications with Twitter activity recorded by both aggregators. Of this overlapped dataset, just 3,135 publications (32.1%) report exactly the same Twitter counts.

**Table 4:** Analysis of (dis)agreement among aggregators in Twitter counts (re)tweets, and distinct tweeters.

	**Altmetric.com** **(n = 17,926)**				**CrossRef ED** **(n = 555)**				**Lagotto** **(n = 9,973)**			
**Tweets**	overlapped	equal	>	<	overlapped	equal	>	<	overlapped	equal	>	<
**CrossRef ED** **(n = 555)**	546	54	8 (5)*	484 (463)*								
**%**		9.9%	1.5%	88.6%								
**Lagotto** **(n = 9,973)**	9,763	3,135	1,027	5,601	515	74	404	37				
**%**		32.1%	10.5%	57.4%		14.4%	78.4%	7.2%				
**Plum Analytics** **(n = 7,526)**	7,356	2402	258	4,696	525	156	355	14	4,143	957	895	2,291
**%**		32.7%	3.5%	63.8%		29.7%	67.6%	2.7%		23.1%	21.6%	55.3%

*The values in the parentheses refer to number of tweeters—only available for Altmetric.com and CrossRef ED.

**Facebook counts**. Regarding Facebook counts, the strongest agreement is between Plum Analytics and Lagotto with 1,130 publications (45.3%) with exactly the same scores (Table 5). The strongest discrepancies are found between Altmetric.com and Plum Analytics, with a total of 1,362 publications (74.9%) with higher scores in Plum Analytics than in Altmetric.com. Plum Analytics also has a total of 1,330 publications (53.3%) with higher scores than in Lagotto. Lagotto has higher Facebook counts in 770 publications (64.5%) with higher scores than in Altmetric.com (Table 5).

**Table 5:** Analysis of (dis)agreement among aggregators in Facebook counts.

	**Altmetric.com** **(n = 3,623)**				**Lagotto** **(n = 2,497)**			
**Facebook counts**	overlapped	equal	>	<	overlapped	equal	>	<
**Lagotto** **(n = 2,497)**	1193	149	770	274				
%		12.5%	64.5%	23.0%				
**Plum Analytics** **(n = 5,149)**	1819	225	1362	232	2496	1130	1330	36
%		12.4%	74.9%	12.8%		45.3%	53.3%	1.4%

**Wikipedia counts**. In terms of Wikipedia citations, the strongest agreement is between Plum Analytics and Altmetric.com as 86.1% of the overlapped publications between them have exactly the same Wikipedia counts. Between Lagotto and Plum Analytics there are 65.6% of overlapped publications with exactly the same Wikipedia counts; while 62.2% of the overlapped publications between Lagotto and Altmetric.com have equal values of Wikipedia counts (Table 6). The lowest agreement is between the Wikipedia counts by CrossRef ED and all other aggregators, although CrossRef ED has systematically higher values of Wikipedia mentions than the other aggregators as showed in Table 2. Hence, the highest discrepancies between the Wikipedia counts reported by CrossRef ED and the others are mostly explained by the higher counts reported in this source.

**Table 6:** Analysis of (dis)agreement among aggregators in Wikipedia counts.

	**Altmetric.com** **(n = 639)**				**CrossRef ED** **(n = 716)**				**Lagotto** **(n = 1,615)**			
**Wikipedia counts**	overlapped	equal	>	<	overlapped	equal	>	<	overlapped	equal	>	<
**CrossRef ED** **(n = 716)**	464	74	367	23								
**%**		15.9%	79.1%	5.0%								
**Lagotto** **(n = 1,615)**	611	380	218	13	643	97	71	475				
**%**		62.2%	35.7%	2.1%		15.1%	11.0%	73.9%				
**Plum Analytics** **(n = 747)**	612	527	42	43	518	97	21	400	697	457	8	232
**%**		86.1%	6.9%	7.0%		18.7%	4.1%	77.2%		65.6%	1.1%	33.3%

#### Correlation among metrics across aggregators

Previous sections have discovered important discrepancies in terms of coverage and counts among altmetric data aggregators. In this section we test the importance of the differences depicted so far using Pearson correlation analyses (Tables 7–10). In order to reduce the effect of publications with zero values in all data aggregators, only publications with at least a non-zero score in any of the aggregators for each of the different social media platforms have been considered.

**Table 7:** Pearson correlation analysis across different aggregators and their Mendeley readership counts.

N = 30,433	**Altmetric.com**	**Lagotto**	**Mendeley**	**Plum Analytics**
**Altmetric.com**	1	.917	.918	.874
**Lagotto**		1	.998	.945
**Mendeley**			1	.946
**Plum Analytics**				1

**Table 8:** Pearson correlation analysis across different aggregators and their tweets and retweets.

N = 18,285	**Altmetric.com**		***CrossRef ED***		**Lagotto**	**Plum Analytics**
	*Tweets*	*tweeters*	*Tweets*	*tweeters*		
**Altmetric.com**						
*Tweets*	1	.979	.636	.602	.952	.762
*tweeters*		1	.593	.578	.955	.752
***CrossRef ED***						
*Tweets*			1	.983	.641	.516
*tweeters*				1	.622	.488
**Lagotto**					1	.728
**Plum Analytics**						1

Tweeters (Twitter users) refer to the number of users who have tweeted publications. This information is available for Altmetric.com and CrossRef Event Data.

**Table 9:** Pearson correlation analysis across different aggregators and their Facebook counts.

N = 6,953	**Altmetric.com**	**Lagotto**	**Plum Analytics**
**Altmetric.com**	1	.112	.134
**Lagotto**		1	.397
**Plum Analytics**			1

**Table 10:** Pearson correlation analysis across different aggregators and their Wikipedia counts.

N = 1,727	**Altmetric.com**	**CrossRef ED**	**Lagotto**	**Plum Analytics**
**Altmetric.com**	1	.380	.551	.867
**CrossRef ED**		1	.276	.388
**Lagotto**			1	.459
**Plum Analytics**				1

**Mendeley readership counts**. As shown in Table 7, a total of 30,433 (96.8%) publications have some readership scores from at least one data aggregator. The correlations among the different aggregators are relatively high (*r>*.*8*) in all cases. The lowest correlations are found between Altmetric.com and the other aggregators. This can be related to the lower coverage of Mendeley readership in Altmetric.com, which does not report Mendeley scores for many publications.

**Twitter counts**. Regarding Twitter counts, correlations vary between high and moderate between most pairs of aggregators. Altmetric.com has the highest correlation with Lagotto (*r* = .*9*) and Plum Analytics (*r* = .*7*) and together with Lagotto they have moderate correlations with CrossRef ED (between *r* = .*5* and *r* = .*6*). Also, there is a moderate correlation (*r* = .*5*) between Twitter users (tweeters) from Altmetric.com and CrossRef ED (Table 8).

**Facebook counts**. Correlations between Facebook counts are low for all pairs of aggregators. The highest correlation is between Plum Analytics and Lagotto (*r* = .*3*) and the weakest correlation is between Altmetric.com and these two platforms (around *r* = .*1*) (Table 9).

**Wikipedia counts**. Correlations for Wikipedia counts range from high (*r* = .*8*) between Altmetric.com and Plum Analytics, moderate (*r = 0*.*5*) between Altmetric.com and Lagotto, to weaker correlation (between *r = 0*.*2* and *r = 0*.*3*) among CrossRef ED and the other three aggregators (Table 10).

Based on the above results, Mendeley counts exhibit the highest correlations. Thus, Mendeley readership counts provided by all data aggregators are relatively consistent, although the coverage is limited in Altmetric.com. Regarding Twitter, correlations are moderate to high (with values ranging between *r* = .*4* and *r* = .*9*). Thus, tweets from Altmetric.com, Lagotto, and Plum Analytics are highly correlated among each other, while the lower correlations are found between CrossRef ED and the other aggregators. Similar levels of correlation between Mendeley readership and tweets across similar altmetric data aggregators have been observed in a previous study for publications from two journals in the Library and Information science field [15]. Regarding Wikipedia counts, Plum Analytics and Altmetric.com are strongly correlated (*r* = .*8*), which is also related to the stronger agreement between these two aggregators in Wikipedia counts. Similar correlations for Wikipedia mentions between Altmetric.com and Plum Analytics have been observed by a recent study for a random sample of 5,000 Web of Science publications from the year 2015 [14]. However, the correlations for Wikipedia counts among the other combinations of aggregators are in general rather weak or just moderate, ranging between *r* = .*2* (for Lagotto and CrossRef ED) and *r* = .*5* (for Lagotto and Altmetric.com). Facebook counts is the source with the lowest correlations overall. Although Facebook counts from Lagotto and Plum Analytics exhibit the highest correlation compared to all other aggregators, the correlation is just of *r* = .*3*. The correlations of Facebook counts with Altmetric.com are in all cases very weak.

### Reasons for differences found across altmetric data aggregators

Although the metrics have been collected at the same time for the same dataset, the results presented above demonstrate that there are relevant differences in the publications covered by the different altmetric aggregators, as well as in the data collected and reported by them. An overview of the main methods of collecting, processing, and reporting altmetric data from the altmetric aggregators considered in this study is presented in Table 11. The information in this table is obtained from the websites of the different aggregators, as well as from the information they have reported in the NISO altmetrics code of conduct [22]. Based on Table 11, in this section, we reflect over the possible reasons for these differences, trying to provide more insights based on additional observations extracted from the data collected. The focus is more on the discussion of the effects of methodological choices than on the benchmark of altmetric aggregators. It is of course very difficult to depict all the underlying reasons for the differences found due to the lack of information on how each aggregator specifically queries and processes the original data sources. However, we argue that most of the data issues identified can be conceptually related to the following four major groups of methodological choices: data collection choices; data aggregation and reporting choices; updating choices; and other technical choices.

**Table 11:** Overview of the main methods of collecting, tracking, and updating metrics across different altmetric data aggregators—As reported by the data aggregators.

Social media sources	Aggregators	Data collection approaches	Data aggregation and reporting approaches			Data updating approaches
		*API use*	*Objects and identifiers*	*Aggregated metrics*	*Raw data & provenance*	*Updates*
**Mendeley Readership**	**Altmetric.com**	Mendeley API.	Tracks, orderly, scholarly objects with DOI, PMID, ArXiv ID and stops the process if any result is found by any of the identifiers.	Aggregated individual user readership counts.	Raw data on readership by academic types, countries, and disciplines is recorded.	Daily updates.
	**Plum Analytics**	Is part of Elsevier and does not directly use the Mendeley API.	Tracks any identifiers (DOIs, PMIDs, etc.)		Raw data is not provided.	
	**Lagotto**	Mendeley API.	Tracks DOIs.	Aggregated individual user and group readership counts.	Raw data is not provided.	
**Twitter**	**Altmetric.com**	Twitter GNIP API.	Tracks a range of different identifiers (URLs, DOIs, PMIDs, ArXiv ids, SSRN IDs, ADS IDs, Amazon URLs, and ISBNs)	Aggregated count of distinct tweeters. Aggregated counts of (re)tweets provided in the Bookmarklet.	Raw data from Twitter (tweets, retweets, tweeters, followers, etc.) is available in the JSON files through the Altmetric.com API	Real-time update.
	**Plum Analytics**		Tracks a range of different identifiers (URLs, DOIs, PMIDs, PMCID, ArXiv IDs, ISBNs, etc. https://plumanalytics.com/identifiers-types-research-output/)	Aggregated counts of (re) tweets across multiple versions of the same output.	Raw data is not provided.	Real-time update.
	**CrossRef ED**	Twitter GNIP Power Track API.	Tracks DOIs and article landing page URLs. No other identifiers.	Only raw data is provided.	Raw data from Twitter (tweets, retweets, tweeters, followers, etc.)	Real-time update.
	**Lagotto**	Twitter Search API with rate limit of 1,800 requests per hour.	Tracks DOIs and journal landing page URLs.	Aggregated counts of (re)tweets.	Raw data is not provided.	No information.
**Facebook**	**Altmetric.com**	Facebook Graph API.	Same as for Twitter.	Aggregated counts of public Facebook posts.	Raw data is not provided.	No information.
	**Plum Analytics**		Same as for Twitter.	Combined counts of all public and private Facebook likes, shares, and comments.		Daily update.
	**Lagotto**		Tracks journal landing page URLs.		Raw data is not provided.	No information.
**Wikipedia**	**Altmetric.com**	Wikipedia API.	Tracks all Wikipedia edits searching for links to scholarly domains, and also clearly labeled identifiers (DOIs and PMIDs).	Count of Wikipedia mentions in the references of English pages based on the 2016 version of altmetric.com.	Raw data is available.	Real-time update.
	**Plum Analytics**	Retrieves Wikipedia mentions by mining search engine results, watching Wikipedia pages for citation changes, and mining the full text of all Wikipedia pages.	Tracks only URLs.	Count of Wikipedia mentions in the references of English pages (Plum Analytics has recently announced the tracking of Spanish and Portuguese language Wikipedia entries: https://plumanalytics.com/spanish-portuguese-wikipedia-references-now-plumx-metrics/).	Raw data is not provided.	Daily update.
	**Lagotto**	MediaWiki API.	Tracks DOIs and URLs.	Aggregated score of mentions in the Wikipedia pages and files.	Wikipedia mentions in the references of the 25 most popular languages of Wikipedia pages (http://www.lagotto.io/docs/wikipedia/) and Wikimedia commons (repository of media files).	No information
	**CrossRef ED**	Wikipedia MediaWiki Event Streams and the MediaWiki APIs.	Only tracks DOIs and article landing page URLs (not any other identifiers).	Count is not provided.	Raw data on Wikipedia mentions in the references of both old and new versions (edits) of English and non-English pages.	Real-time update.

#### Data collection choices

Metrics depend largely on the way each aggregator collects the data from the related social media sources. This can be done directly from the original social media platform, or indirectly through a third-party vendor, bot, or agent [25]. In this case, the use of different APIs can partly explain the differences in the values of metrics reported by the altmetric aggregations. Additionally, the focus on different identifiers, URLs, landing pages, or scholarly objects can also provide different results.

**Mendeley**. Except Plum Analytics, Altmetric.com and Lagotto use the same Mendeley REST API. However, the specific way each aggregator queries the API using specific or multiple identifiers can have an effect on the final reported readership counts. For instance, Altmetric.com only queries Mendeley readership when other altmetric event has been reported for the publication [26; 27]. This largely explains the strong discrepancies between this aggregator and the others in the reporting of Mendeley readership. Also, Altmetric.com queries Mendeley first using the DOI of the publication, and if no record is found then the PMID, and subsequently the ArXiv id are used. It stops the API when one of the identifiers has provided a match. Lagotto queries Mendeley API using only DOIs. In contrast, Plum Analytics does not query Mendeley API since it gets the data from Mendeley as it is part of Elsevier. Plum Analytics cross-reference all identifiers for a given object against data that they get from Mendeley and provides aggregated readership counts for all versions of the same object.

Hence, these different approaches can explain why Altmetric.com has a much lower coverage of publications with Mendeley readership, although its agreement with our DOI-approach method for Mendeley is strong. In contrast, Plum Analytics exhibits higher values and coverage, but has lower agreement with other aggregators.

**Twitter**. The collection of tweets using the Twitter Search API differs significantly from using the Streaming APIs (see S1 File for an explanation of the differences between them, also see https://dev.twitter.com/rest/public/search). The use of Twitter APIs provided by third-party companies such as GNIP could also influences the metrics provided by the different altmetric aggregators. For example, GNIP offers access to real-time data with the possibility of filtering the searches based on keywords, geo-locations, etc. This in turn requires payment and depends on GNIP’s sale agreement with the social media source (Twitter). Moreover, the frequency and the type of query used to use the Twitter API by the altmetric aggregators, also influence the metrics provided by them [28].

Most of the analyzed altmetric aggregators use GNIP for collecting tweet mentions, except Lagotto that uses the Twitter search API with a rate limit of 1,800 queries per hour (http://www.lagotto.io/docs/twitter_search/). However, altmetric aggregators also query differently the Twitter API (see S1 File for the methodological descriptions of Altmetric.com, CrossRef ED, Lagotto, and Plum Analytics). Unfortunately, there is no direct way to explore how each aggregator exactly uses these third party APIs and how their algorithms collect and process the metrics. Based on this, we can just indirectly conclude that the choice of the Twitter API with rate limit could explain both why Lagotto reports substantially lower scores in the overlapped publications with both Altmetric.com and Plum Analytics. Additionally, the focus on mentions to just DOIs and journal landing page URLs for the registered contents by CrossRef ED can explain the lower Twitter coverage and counts provided by this aggregators in contrast with other aggregators (Altmetric.com and Plum Analytics) that typically also use other identifiers (e.g. PMID, ArXiv id, etc.) to track the mentions to the papers. Also the recent start of CrossRef ED may imply that they have started to collect tweets from their inception but not retrospectively, thus explaining why the other altmetric aggregators exhibit so much higher Twitter counts. Furthermore, how each aggregator accommodates the Twitter compliance guidelines also influences the data reported for each tweet. For instance, if a user deletes a tweet, changes its sharing options from public to protected or withheld, or has their account deleted or suspended, the tweet will be immediately removed and will not be displayed by Plum Analytics (which would imply a decrease in the number of tweet counts for the publication)(http://support.gnip.com/apis/consuming_compliance_data.html). In contrast, Altmetric.com reports the deleted tweets but doesn’t display the tweets that are no longer public (which implies that the count won’t decrease but wouldn’t be possible to fully recreate it) (http://support.gnip.com/apis/consuming_compliance_data.html).

**Facebook counts**. All the three aggregators that collect Facebook mentions (Altmetric.com, Lagotto, and Plum Analytics) use the same Facebook Graph API. However, with respect to the choice of identifiers, Lagotto uses journal landing page URLs while Plum Analytics and Altmetric.com use any identifiers for collecting Facebook mentions (same approach as for Twitter). Hence, the use of additional identifiers by Plum Analytics explains the higher Facebook counts than Lagotto. Furthermore, the choice of aggregators in collecting public or private scores also results in variations in the metrics offered by them. In this case, Lagotto and Plum Analytics collect both Facebook public and private posts while Altmetric.com collects only public wall posts. Additionally Plum Analytics and Lagotto also count other Facebook events such as likes, shares, and comments. These choices explain the substantially lower Facebook coverage and values reported by Altmetric.com compared to Lagotto and Plum Analytics.

**Wikipedia counts**. With respect to Wikipedia mentions, the choice of API also differs across aggregators. For example, Altmetric.com uses the Wikipedia API, Lagotto uses the MediaWiki API, CrossRef ED uses the MediaWiki Event Streams and the MediaWiki API. In contrast, Plum Analytics retrieves Wikipedia mentions by combining different methods of mining search engine results, the full text of all Wikipedia pages, and looking into Wikipedia pages for citation changes. Clearly, all these approaches could contribute to explain the differences in the Wikipedia counts reported by these aggregators. Moreover, other reasons for the observed differences relate to the choice of aggregations are explained in the next section.

#### Data aggregation and reporting choices

Metrics can largely be influenced by how scores for different versions of the same object with possible different identifiers (DOI, PMID, ArXiv ID, URL) or versions (e.g. the ArXiv versions, the published version) are aggregated as well as how different events coming from the same social media platform are combined and reported. Moreover, aggregations could be based on different languages, edits, types, and scholarly objects.

**Aggregations of different identifiers and versions of the same publication**. The metrics calculated for a publication may also depend on the quality of the metadata for which social media events are collected as well as on the existence of duplicate records of the same publication. Differences in metrics may arise when altmetric data aggregators handle differently these duplicates and merge (or not) different scores coming from multiple identifiers or versions of the same object.

**Fig 1:**
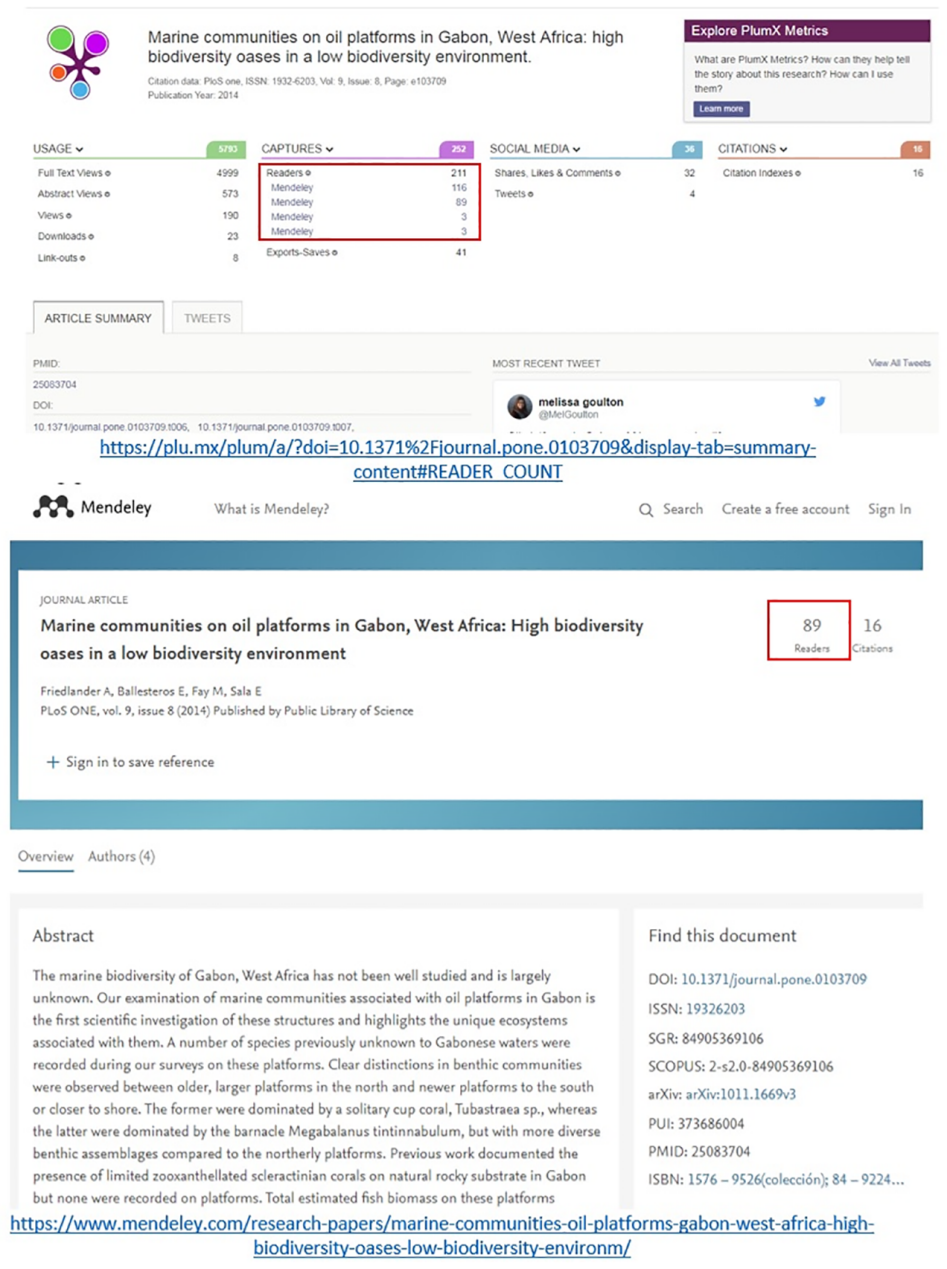
Examples of different readership counts across different altmetric aggregators: Plum Analytics vs. Mendeley (accessed on 15 December 2017).

Both Lagotto and Plum Analytics provide the largest coverage of publications with at least one reader and the highest counts of Mendeley readership. A potential explanation for these higher counts can be the merging of counts from different identifiers (e.g. DOI and PMIDs) for the same publication. This seems to be the case for Plum Analytics (see S1 File). The problem is that the merging of counts from different identifiers can also imply some degree of error. For example, wrong linkages between identifiers happen and may create over or under-merging of records. Considering that Mendeley is a user-driven database, users may create wrong linkages between PMIDs, DOIs, etc. and therefore this sometimes causes errors in the assignment of readership to publications. Fig 1 shows how the linkages to both wrong DOIs and PMIDs in Mendeley leads to higher Mendeley readership reported by Plum Analytics. Plum Analytics aggregates readership (211 reader counts instead of 89 reader counts from Mendeley) for the paper with both correct and incorrect DOIs and PMIDs in Mendeley (See S2 File) and thus, incorrectly reports 122 higher reader counts.

**Fig 2:**
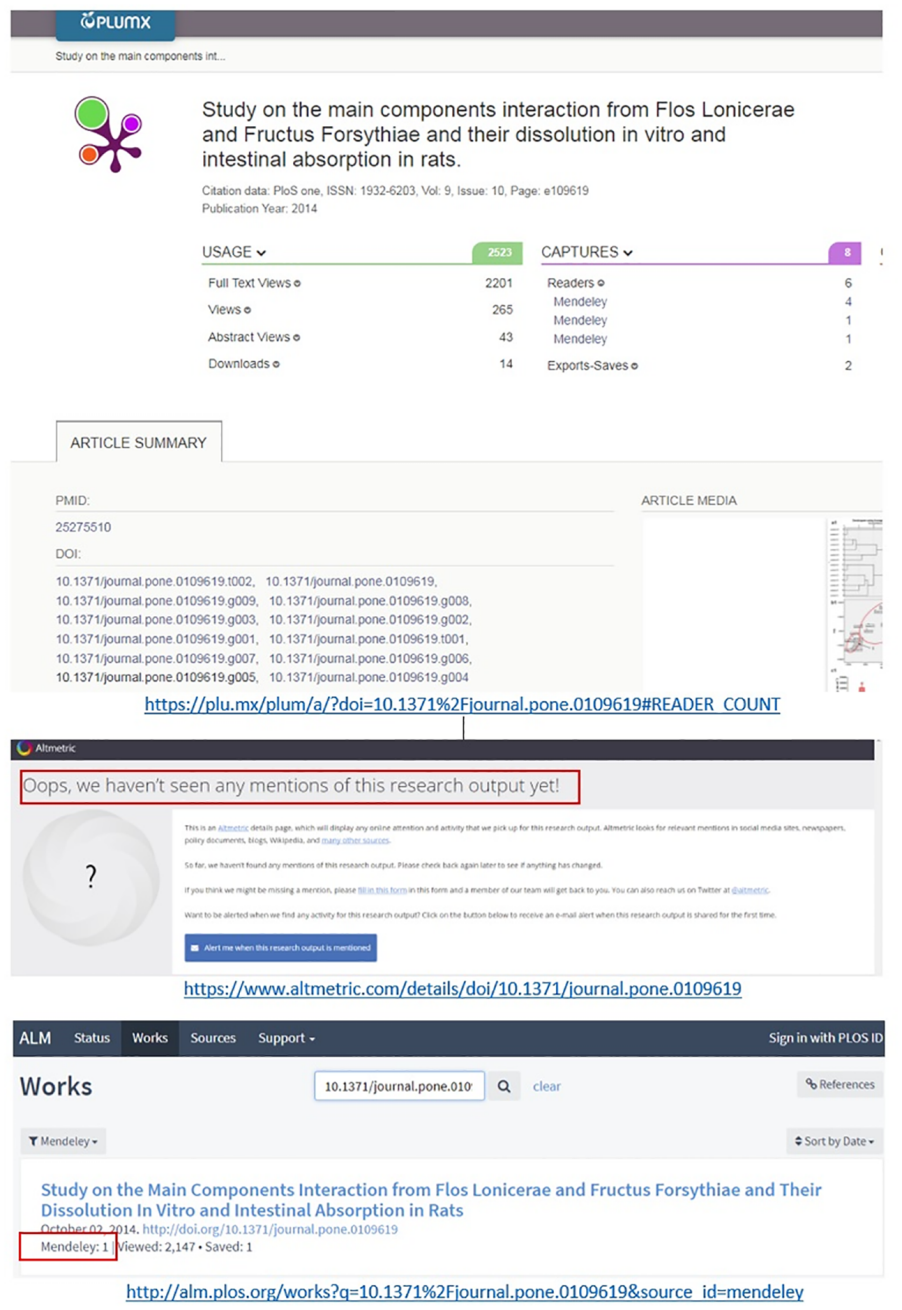
Examples of different readership counts across different altmetric aggregators: Plum Analytics, Altmetric.com, and Lagotto vs. Mendeley (accessed on 15 December 2017).

Also, when the same object appears with different records in Mendeley, for example one with a DOI and another one with a PMID, Plum Analytics aggregates all the readership counts across all the different versions. Fig 2 provides an example of how Plum Analytics aggregates Mendeley readership counts (6 counts in total across 2 versions) for all the multiple versions (duplicate records) in Mendeley of the same object. This example illustrates how wrong linkages to PMID in Mendeley affects the total readership counts reported. The readership counts across three different versions of the same object is reported in Fig 2. The record with 4 counts is recorded with both DOI and PMID while the other two records with 1 count each have only DOI or only PMID in Mendeley (See S2 File). However, the record with PMID in Mendeley has a wrong linkage to PMID and this leads to incorrectly reporting one extra readership count by Plum Analytics. Lagotto finds the record with only 1 readership for this object and fails to report the record with 4 readerships in Mendeley. Altmetric.com fails to find any readership for the same object since it is not mentioned in any other sources it tracks.

**Aggregations of different events from the same social media platform**. Aggregators may use the same API to query some data sources such as Twitter, Facebook, or Mendeley (i.e. Twitter GNIP API, Facebook Graph API, or Mendeley API); however, they could differ in the ways they combine different forms of scores from the same social media platform.

**Fig 3:**
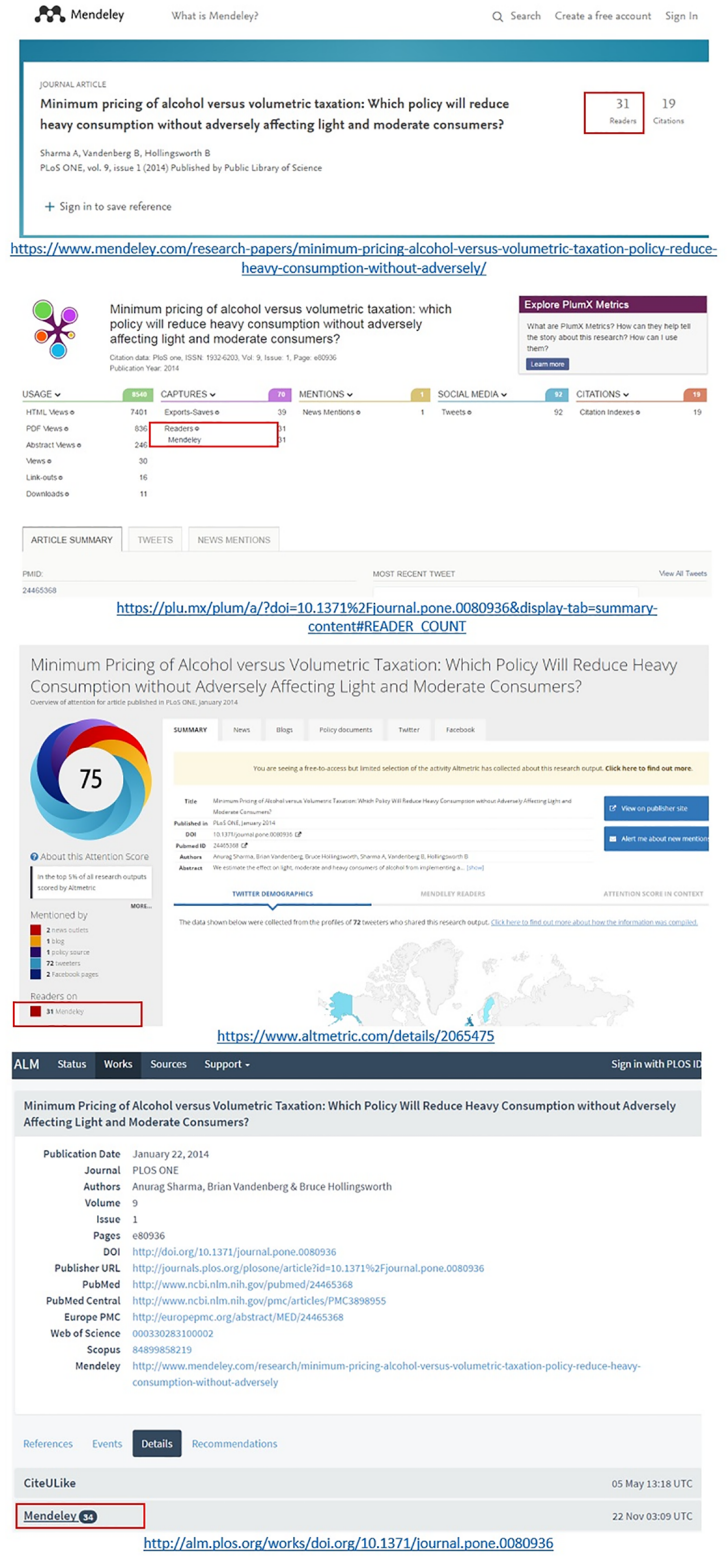
Examples of different Mendeley readership counts across different altmetric aggregators: Mendeley, Plum Analytics, Altmetric.com, and Lagotto are presented orderly (accessed on 29 November 2017).

**Mendeley**. The higher readership values reported by Lagotto in comparison to the other aggregators can be explained by Lagotto’s choice of reporting combined readership values of individual user and group counts. As an example in Fig 3, a publication with 31 readership according to Mendeley is presented, for which Lagotto reports 31 readers, plus three “group count” readers, making a total of 34; while Altmetric.com and Plum Analytics both report 31 readers each.

**Fig 4:**
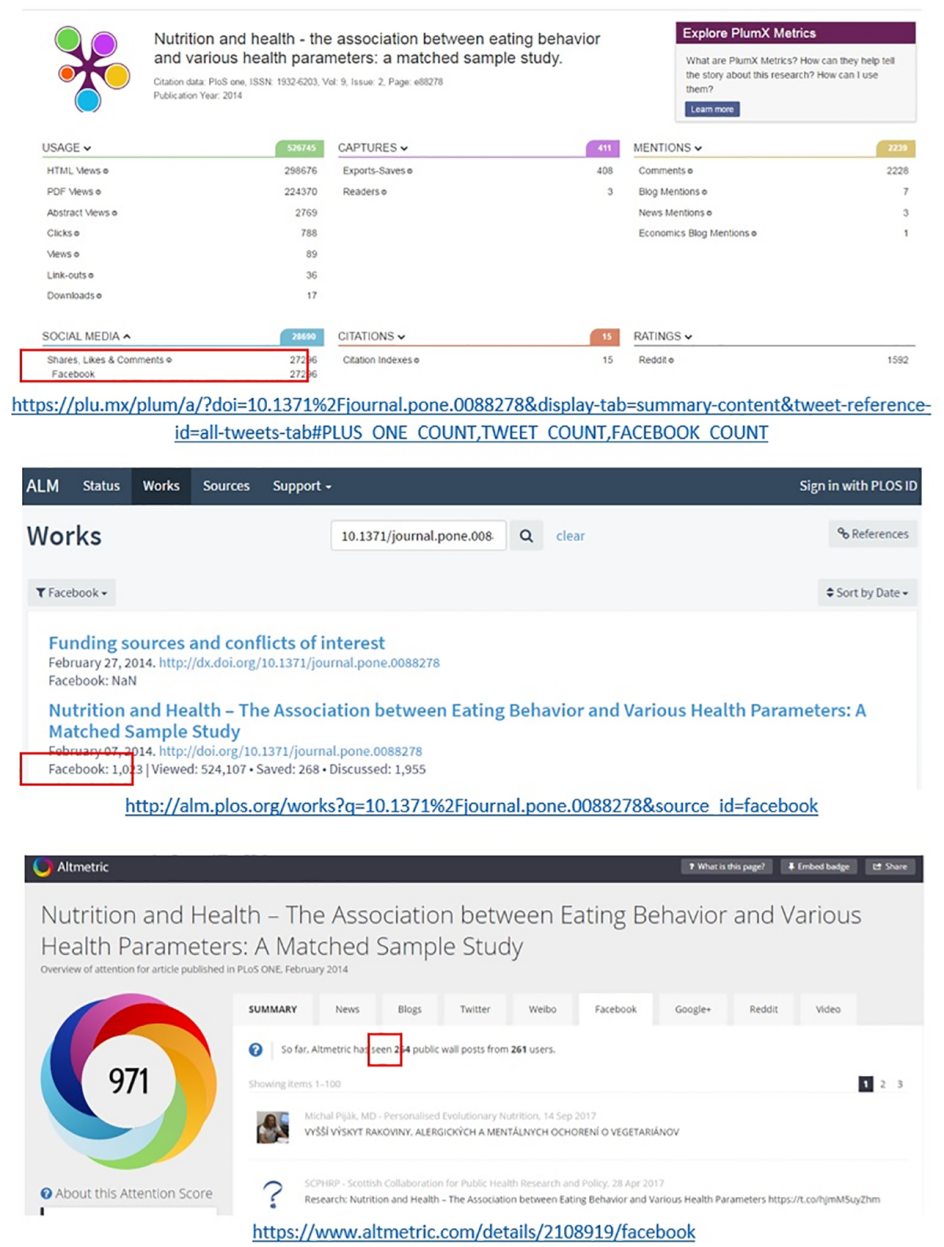
Examples of different Facebook counts across different altmetric aggregators: Plum Analytics, Lagotto, and Altmetirc.com are presented orderly (accessed on 29 November 2017).

**Facebook**. Fig 4 illustrates an example of large differences in Facebook counts. Lagotto reports a total 1,023 combined Facebook score of all activities (posts, shares, likes, comments), while Altmetric.com reports 264 Facebook public post counts (excluding likes, individual timeline, and private posts). Plum Analytics exhibits the largest number of Facebook counts (27,296) including the sum of all public and private Facebook likes, shares, and comments across the multiple identifiers that it tracks for the same publication. Unfortunately the lack of access to the raw data does not allow exploring further the reasons for such large difference between Lagotto and Plum Analytics.

**Twitter**. Most of the altmetric aggregators report different indicators on Twitter events. For example, in case of Altmetric.com and Lagotto, both tweets and retweets are combined for each publication (although the indicator promoted by Altmetric.com is the number of distinct tweeters, both from tweets and retweets). Plum Analytics reports separately the number of tweets and retweets that mention the object, but it combines both counts in a final Twitter score in the main summary. Plum Analytics also reports combined scores of (re)tweets for the different versions of the same object. CrossRef ED also reports all data of tweets and retweets. The higher value of tweets reported by Altmetric.com compared to other aggregators could be explained by the fact that “Altmetric.com collates the scores for the different version of the same research output” (see S1 File). However, it is not clear why CrossRef ED provides the least tweets than other aggregators. A potential explanation is that as publications from 2014 analyzed in this study while CrossRef ED has started in April 2017, tweets between 2014 and 2017 could be missing.

**Fig 5:**
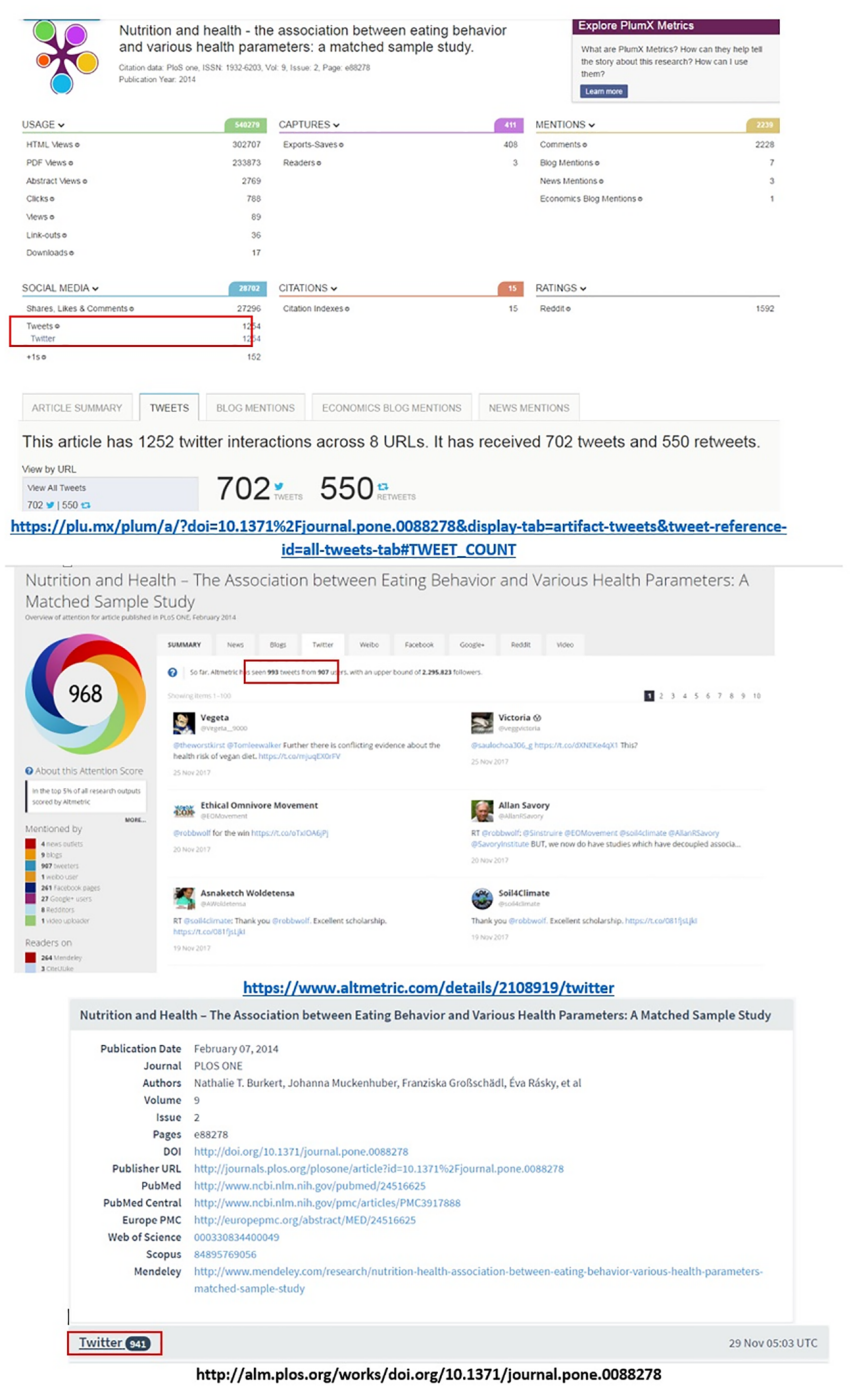
Examples of different tweets (tweeters) across different altmetric aggregators: Plum Analytics, Altmetric.com, and Lagotto are presented orderly (accessed on 29 November 2017).

Fig 5 illustrates an example of the Twitter counts from these aggregators for the same publication. Overall, Plum Analytics reports 1,254 (702 tweets and 550 retweets) across the 8 different URLs pointing to the same publication from different databases such as PubMed, PMC, and PloS ONE. Hence the tweet value is higher than the values reported by Altmetric.com (907) and Lagotto (941) while CrossRef ED reports no tweets for this publication.

**Aggregation based on languages, document types, scholarly objects, and edits**. The choice of aggregators in aggregating scores for particular data sources, document types, languages, or scholarly objects could also influence the metrics provided. For instance, discrepancies in the value of Wikipedia mentions can be explained by the different approaches in aggregating DOIs mentioned across Wikipedia pages in different languages as well as non-encyclopedia pages (such as user, talk, and Meta-wiki pages, media and files). Also, consideration of the edits of each Wikipedia page as separate events influences the counts.

**Fig 6:**
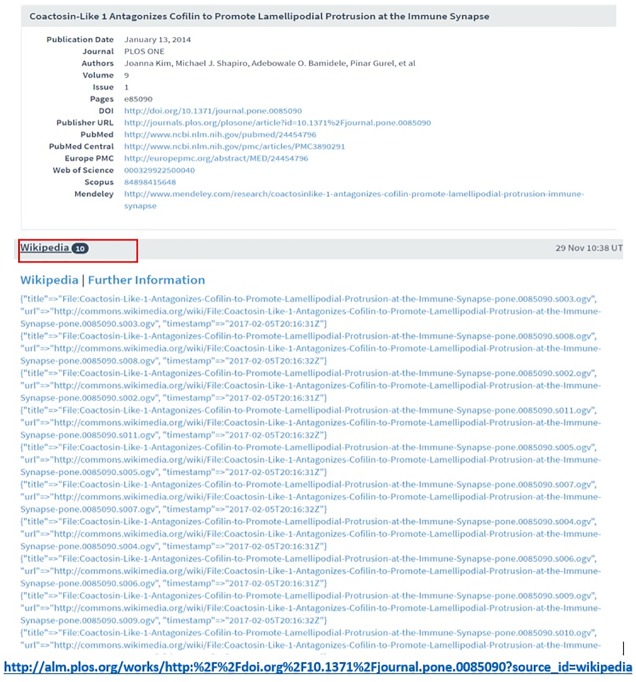
Examples of Wikipedia counts for an object reported by Lagotto (accessed on 29 November 2017).

For instance, the Wikipedia mentions count reported by Lagotto is a combined score of the number of references to papers, material files, images, etc. from the 25 most popular Wikipedia sites (http://www.lagotto.io/docs/wikipedia/). In contrast, Altmetric.com reports Wikipedia mentions of scholarly outputs collected from the reference sections of English, and from 2017 onwards Finnish, and Swedish languages Wikipedia entries. Altmetric.com doesn’t track non-encyclopedic pages (https://help.altmetric.com/support/solutions/articles/6000060980-how-does-altmetric-track-mentions-on-wikipedia-). For Altmetric.com also every Wikipedia mention has to have an author, a timestamp, and valid citations such as title, PubMed ID, or DOI to be tracked. Wikipedia mentions reported by CrossRef ED includes ‘edits of articles in Wikipedia’ and combines mentions from all old and new versions (https://en.wikipedia.org/w/index.php?title=Finger&oldid=783699149), thus every edit of the paper is considered separately. This explains the higher Wikipedia mentions recorded by CrossRef ED. However, the approach taken by Plum Analytics for tracking Wikipedia mentions is different from the others. Plum Analytics uses a combination of data mining both in the search engine results and in open source repository platforms (such as Dspace http://www.dspace.org/introducing) and watches citation changes in the Wikipedia pages. It mines full text of Wikipedia English pages (from March 2018 onwards Spanish and Portuguese are also tracked) and looks for any links to the object (DOIs, PMIDs, URLs). Also, Plum Analytics tracks scholarly objects (thesis, book chapters, books, and technical reports) other than articles (http://plumanalytics.com/wikipedia-altmetrics-calculating-mention-metrics/). The example in Fig 6 shows that Lagotto reports 10 Wikipedia mentions while the other aggregators do not report any for the same publication. This is because Lagotto, besides papers, also tracks files that contains links to papers, while the other aggregators don’t do that.

**Fig 7:**
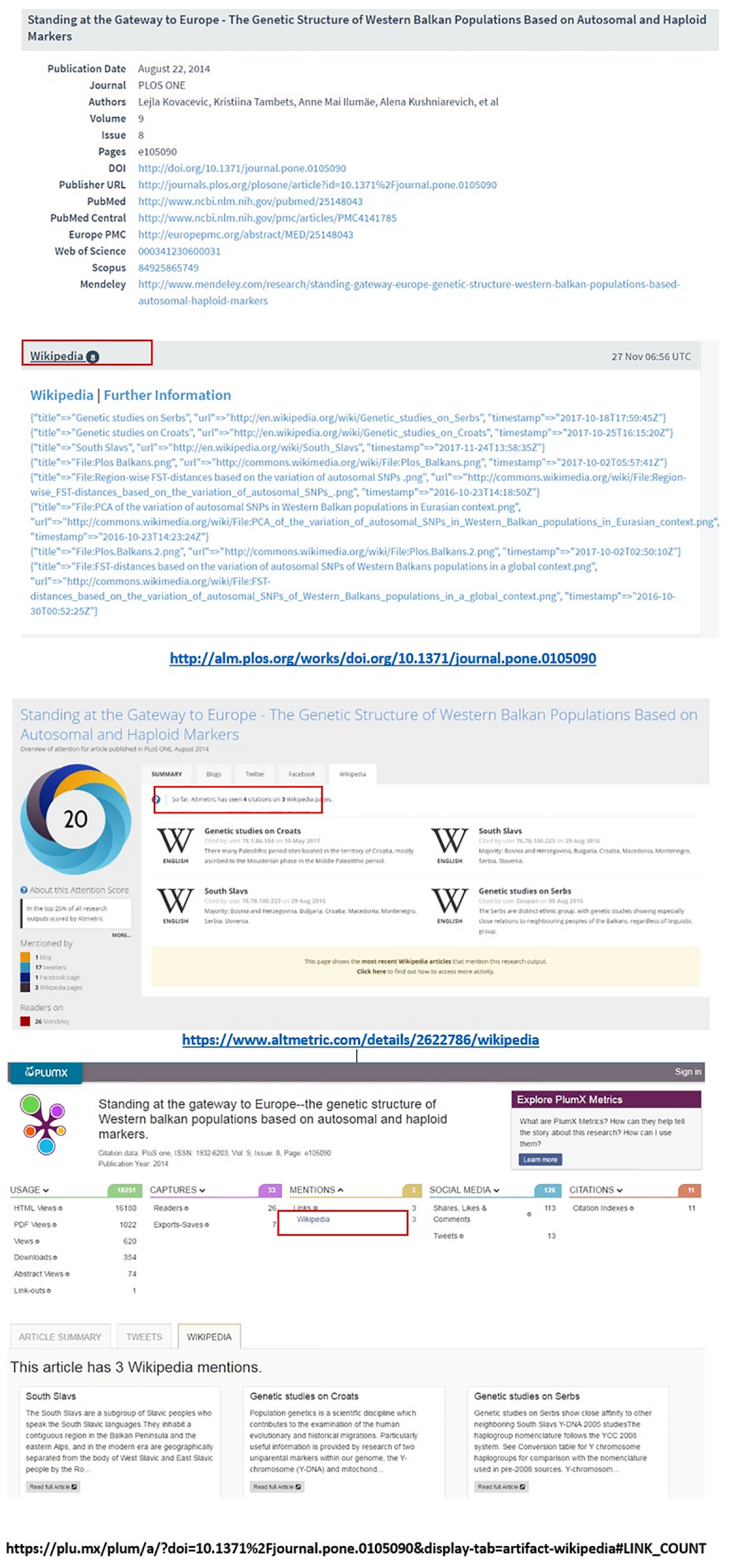
Examples of different Wikipedia mentions across different altmetric aggregators: Lagotto, Altmetric.com, and Plum Analytics are presented orderly (accessed on 29 November 2017).

The example depicted in Fig 7 shows a paper for which Lagotto reports 8 mentions (three in English language Wikipedia pages + 5 files). Altmetric.com reports 3 mentions (actually 4 citations found on 3 English language pages, but Altmetric.com only counts the number of distinct Wikipedia pages citing the publication). Plum Analytics also reports 3 mentions (in 3 English language pages) while CrossRef ED records 315 mentions (English and Macedonian language pages + edits made at different times) (See S3 File).

**Form of updates**. It is not possible to know how exactly each aggregator queries the original social media sources and how often they update their data, besides the information reported by them. However, it is technically possible that differences in the date and time when social media events occurred and when the aggregator collected them, together with time lags in the frequency of updates of each aggregator, also cause discrepancies in the metrics provided by each aggregator. Although it is assumed that in most cases all aggregators have updated their platforms in real time or, depending on the data source, on a daily basis (as presented in Table 11), in most cases the information on the exact time of update across different aggregators is not available. Another reason for discrepancies in the updates of Mendeley metrics includes the time lags between the actual act of saving a paper by a Mendeley user, the update of the Mendeley readership of the publication by Mendeley and the moment when the aggregators collect their data. Moreover, the periodical update of the Mendeley database which only happens instantly for readership counts but periodically for their decrease (www.niso.org/apps/group_public/view_comment.php?comment_id=632 and http://www.niso.org/apps/group_public/view_comment.php?comment_id=610) could also contribute to explain some of the differences found (see S1 File).

**Other technical reasons**. Other technical issues include the matching rate of identifiers with journal publisher’s platforms and their policy in allowing access, API speed, and rate of querying. Metrics depend on the matching rate of DOIs and URLs of an object by aggregators. There are differences across journal publisher platforms in resolving DOIs to journal landing pages. Whether a publisher allows DOIs resolving and how simple is this process (cookies problems, access denies, redirects) depend on the publishers’ policies [29]. Hence, differences in metrics including any possible agreements between altmetric aggregators and specific publishers result in their different coverage of publications from different publishers [30]. For instance, whether all the variations of journal publisher’s URLs for a given DOI is known by the aggregator or not and the extent to which an aggregator is able to call the provider’s API (for example Facebook API) for a given DOI to cover all the mentions across multiple URLs could influence aggregator’s coverage of different publishers. Plum Analytics and Lagotto both track all the possible URLs for a given DOI from different publishers in both public and private posts. Thus, this can also contribute to the highest Facebook counts reported by Plum and Lagotto. Other issues such as availability of different ranges of identifiers (DOIs, PubMed, SSRN, ArXiv IDs, etc.) tracked, how shortened URLs are handled, how rate limits of data aggregator and third party provider APIs are handled, or the functioning of the rate of traffic over the API, are all technical issues that could influence the rate of querying APIs (http://www.eventdata.crossref.org/guide/sources-in-depth/) and hence could also influence the metrics provided by the aggregators.

## Conclusions

The proliferation of new social-media-based indicators has opened the possibility to study the interactions between social media and science in what can be seen as the social media studies of science [1;2]. However, the development of these studies has a strong dependency on the specific data and metrics available. Several grand challenges have been already pointed out regarding the development and potential applicability of these new data sources. “Heterogeneity”, “data quality”, and external “dependencies” have been argued as major challenges of altmetric data [3]. In this study, we specifically focus on the challenge related with “data quality” (although to some extent we also exemplify some of the “external dependencies” involved). Social media metrics data collection relies on a large range of different methodological and technical choices (e.g. APIs, identifiers tracked, forms of querying original sources, types of events recorded, selections of publishers) and reporting choices (e.g. aggregation of different types of counts into one single metric, grouping of different metrics into broader categories, combination of different counts for different identifiers). Hence, it is important to understand how these choices may affect the data collected and reported by different aggregators.

This study describes how the social media metrics collected for a same set of DOIs at the same time may vary across different major altmetric aggregators. Similar results have been found in recent studies comparing different altmetric data aggregators [14;15;21] as well as other previous studies [11; 13; 16; 17]. For instance, the same high consistency across aggregators regarding Mendeley readership has been highlighted in these previous studies.

More specifically, our results showed that Lagotto and Plum Analytics provide the highest values of Mendeley readership. This can be explained by the choice of aggregating the counts coming from different identifiers of the same paper, or the different consideration of forms of readership (e.g. individual readership and group readership). Altmetric.com provides the highest value of tweets, which could be explained by the tracking and combination of counts from different versions of the same object. Plum Analytics provides the highest value of Facebook counts as it combines different events from Facebook in the same score, and CrossRef ED provides the highest value of Wikipedia mentions, as it collects mentions from different languages and edits of the same Wikipedia entry. Correlation analysis showed that the differences across aggregators for Mendeley readership counts are the least problematic, since the different values tend to correlate quite strongly (although the limited coverage of Altmetric.com with respect to Mendeley readership needs to be reminded. Although some relatively moderate correlations found across some data aggregators (particularly for CrossRef ED data with the other aggregators), the overall correlation analyses of Twitter counts suggest a reasonably good agreement among data aggregators. The lowest correlations among aggregators are found for Facebook and Wikipedia counts. For these sources it seems that the choices adopted by each of the aggregators in collecting and processing the counts have a strong relevance on the final counts reported by them. For these two sources, it is important for the users to understand what the aggregators are actually computing.

Overall we can argue that most of the differences found across data aggregators are explained by specific choices on the data collection and aggregation approaches as explained here. All of these choices can have different effects on the results and analytical approaches based on altmetric data. For example, the choice of aggregating all Mendeley readership from the different version of the same paper may have an inflationary effect. This inflationary effect can be challenging when the pairing of document identifiers is wrong (e.g. users wrongly linking DOIs and PMIDs [see Figs 1 and 2]). The choice of counting together different acts from the same social media source, like tweets or retweets, has also conceptual repercussions, since a tweet can be seen as an act of greater engagement [31] than a retweet. This can also be argued for the combined count of Facebook posts, shares, likes, etc., which breaks the internal homogeneity of the indicator [1]. This hinders the interpretability and meaning of the indicator, and opens the possibility for its easier manipulation. In a similar fashion, the counting of Wikipedia mentions of different edits of the same Wikipedia entry has conceptual issues. The consideration of some different language versions of the same Wikipedia entry may be tricky, creating biases favoring publications form the countries of these languages (e.g. Finland and Sweden in the case of Altmetric.com Wikipedia counts and Spanish and Portuguese in the case of Plum Analytics Wikipedia counts). Also, the mere translations of a Wikipedia entry may derive in multiple mentions for a publication without reflecting a real engagement from the translators with the cited publications. Wikipedia articles are often translated by bots or applications (https://en.wikipedia.org/wiki/Swedish_Wikipedia) and hence it doesn’t always reflect the engagement of the translator with the content.

All in all, it is difficult to claim that some choices are better than others and hence the solution to the discrepancies among different aggregators cannot just be solved by recommending to use those that provide the higher counts. Actually, our results suggest that it is difficult to come up with universal recommendations on what aggregators must choose. All of them exhibit advantages and disadvantages depending on the choices and sources tracked. At best, we can talk about *overall recommendations* for both data aggregators and social media metrics users:

1. *Increase the transparency around the methodological choices in data collection*. Aggregators use different strategies in collecting, calculating, or updating metrics. These strategies may involve technical issues such as linking duplicate records or merging different acts from the same platform in one count. In this sense, although the efforts of data aggregators for making better aggregations of metrics for publications is commendable, it is critical that they are more transparent on how they have collected and aggregated the data. Users, should also be aware that these choices may imply potential risks for their analysis, and should demand more and transparent explanations on these for the analytical use of the data.
2. *Increase the awareness of unintended effects of methodological choices*. Both users, researchers, and data aggregators should be aware of the unintended effects that methodological choices can have in the use and application of social media metrics data and its application. For instance, tracking mentions to the publisher’s URL (in addition to some other identifiers) may increase the counts to publications from some publishers (which are tracked) but miss those of other less known publishers, thus creating a bias towards the tracked publishers. Hence, it could be argued that Crossref ED approach of just tracking registered DOIs could be seen as a less biased approach, although it provides lower Twitter scores than other aggregators. Similarly, if some metrics or counts are dependent on the occurrence of other events (e.g. Mendeley counts in Altmetric.com) this can also have effects on the analytical validity of this data source depending on the objectives of the user. These potential biases and limitations should be explained and users must be aware of them.
3. *Increase the transparency around the computation of different social media acts*. Disclosing the combined computation of different social media acts into one metric is also important (e.g. whether Facebook or Twitter counts include posts, likes, shares, comments). This is critical in order to understand the internal homogeneity and conceptual value of the metrics reported. Considering the infancy of social media metrics and the uncertainty in the relationship and meaning of the different social media events, it seems reasonable to argue that keeping events separate as much as possible is probably the best approach from an analytical perspective. The combination of conceptually different metrics into one single measure may introduce misunderstandings, misuses, and even manipulations that could have negative effects on the further application of social media metrics.
4. *Increase the replicability and interactivity of the data reported*. Recording, disclosing, and making available the original raw social media data to the users would allow them to make their own choices, as well as to obtain a better idea of the origin and provenance of the data collected. As emphasized by the *NISO altmetrics data quality Code of Conduct*, documenting the degree of transparency on how each aggregator queries different data sources and the processes taken, is to be preferred in order to make it possible to verify the metrics. Also, providing some information on the accessible content by the different data sources, characteristics of their underlying data, internal processes applied by aggregators, how they access external sources, and their strategy to calculate or report metrics, helps to gain better understanding of the relevant issues faced by each aggregator when collecting and processing social media data. From the user point of view, it is recommended to demand more interactive possibilities when it comes to the use and analysis of social media metrics. Hence, incorporating analytical features through which users can choose sources, periods of time, types of social media acts as well as indicators, can help to empower the user in the application, replication, and better interpretation of social media metrics.

The results of this paper provide some original insights about current data challenges in social media metrics. It is important to emphasize that the validity and reliability of social media metrics sources should be constantly checked and discussed, particularly among altmetric data aggregators, researchers in social media metrics, and the users of this data. The importance of these methodological choices in data collection and calculation of metrics should be incorporated in the overall discussion around social media metrics research. Understanding how methodological and technical choices can influence the analytical reliability and validity of social media metrics is a critical element in the future development of social media studies of science. Future research should also focus on providing further insights and possible solutions for current and potential data challenges in social media data collection. Also, other suggestions for future studies could include whether the choice of journals (open access vs. closed) affects the results obtained in the current study. Moreover, the extent to which each aggregator combines metrics for the different versions of publications, different identifiers of the same object, and how does these combinations influence the metrics provided by the aggregators needs to be further studied.

## Supporting information

S1 File Extracts of methodological descriptions from Social Media platforms and Altmetric aggregators. (PDF)

S2 File Examples of records in Mendeley with incorrect PMIDs. (PDF)

S3 File Excerpt of the JSON file from CrossRef ED recording 315 Wikipedia mentions for an object with DOI: 10.1371.journal.pone.0105090). (PDF)

S4 File List of DOIs and their social media metrics used in this study: https://doi.org/10.6084/m9.figshare.6061262.v1. (XLSX)

## References

[1] Wouters P., Zahedi Z., & Costas R. (2018). Social media metrics for new scientific evaluations In Glänzel W., Moed H. F., Schmoch U., & Thelwall M. (Eds.), Handbook of Quantitative Science and Technology Research. Springer.

[2] Costas R. (2017). Towards the social media studies of science: social media metrics, present and future. *Bibliotecas* Anales de Investigación, 13(1), 1–5.

[3] Haustein S. (2016). Grand challenges in altmetrics: heterogeneity, data quality and dependencies. Scientometrics, 108(1), 413–423.

[4] Halevi G., Moed H., & Bar-Ilan J. (2017). Suitability of Google Scholar as a source of scientific information and as a source of data for scientific evaluation? Review of the Literature. Journal of Informetrics, 11(3), 823–834. doi: 10.1016/j.joi.2017.06.005

[5] Moed H. F., Bar-Ilan J., & Halevi G. (2016). A new methodology for comparing Google Scholar and Scopus. Journal of Informetrics, 10(2), 533–551. doi: 10.1016/j.joi.2016.04.017

[6] Delgado López-Cózar E., Robinson-García N., & Torres-Salinas D. (2014). The Google scholar experiment: How to index false papers and manipulate bibliometric indicators. Journal of the Association for Information Science and Technology, 65(3), 446–454. doi: 10.1002/asi.23056

[7] Olensky M., Schmidt M., & van Eck N. J. (2016). Evaluation of the citation matching algorithms of CWTS and IFQ in comparison to the Web of science. Journal of the Association for Information Science and Technology, 67(10), 2550–2564. doi: 10.1002/asi.23590

[8] Franceschini F., Maisano D., & Mastrogiacomo L. (2016). The museum of errors / horrors in Scopus. Journal of Informetrics, 10, 174–182. doi: 10.1016/j.joi.2015.11.006

[9] Valderrama-Zurián J. C., Aguilar-Moya R., Melero-Fuentes D., & Aleixandre-Benavent R. (2015). A systematic analysis of duplicate records in Scopus. Journal of Informetrics, 9(3), 570–576. doi: 10.1016/j.joi.2015.05.002

[10] Jacsó P. (2011). Google Scholar duped and deduped: the aura of “robometrics”. Online Information Review, 35(1), 154–160. doi: 10.1108/14684521111113632

[11] Zahedi, Z., Fenner, M., & Costas, R. (2015). Consistency among altmetrics data providers/aggregators: What are the challenges? In altmetrics15: 5 years in, what do we know? Amsterdam, The Netherlands. Retrieved from http://altmetrics.org/wp-content/uploads/2015/09/altmetrics15_paper_14.pdf

[12] Zahedi, Z., Bowman, T. D., & Haustein, S. (2014a). Exploring data quality and retrieval strategies for Mendeley reader counts. Presented at the SIG/MET workshop, ASIS&T 2014 annual meeting, Seattle. Retrieved from: www.asis.org/SIG/SIGMET/data/uploads/sigmet2014/zahedi.pdf

[13] Chamberlain S. (2013). Consuming article-level metrics: Observations and lessons from comparing aggregator providers data. Information Standards Quarterly, 25 (2), 4–13.

[14] Meschede C., & Siebenlist T. (2018). Cross-metric compatability and inconsistencies of altmetrics. Scientometrics. http://doi.org/10.1007/s11192-018-2674-1

[15] Bar-Ilan, J. & Halevi, G. (2017). Altmetric Counts from Different Sources: A Case Study of Journal of the Association for Information Science and Technology (JASIST) Articles Published Between 2001 and Mid 2017. The 2017 Altmetrics Workshop: The Dependencies of Altmetrics, 26 September 2017, Toronto, Canada. Retrieved from: http://altmetrics.org/altmetrics17/

[16] Zahedi, Z., Fenner, M., & Costas, R. (2014). How consistent are altmetrics providers? Study of 1000 PLOS ONE publications using the PLOS ALM, Mendeley and Altmetric.com APIs. In altmetrics 14. Workshop at the Web Science Conference, Bloomington, USA.

[17] Jobmann A., Hoffmann C. P., Künne S., Peters I., Schmitz J., & Wollnik-korn G. (2014). Altmetrics for large, multidisciplinary research groups: Comparison of current tools. Bibliometrie—Praxis Und Forschung, 3(1), 1–19.

[18] Harzing A. W., & Alakangas S. (2016). Google Scholar, Scopus and the Web of Science: a longitudinal and cross-disciplinary comparison. Scientometrics, 106(2), 787–804. doi: 10.1007/s11192-015-1798-9

[19] Meho L. I., & Sugimoto C. R. (2009). Assessing the scholarly impact of information studies: A tale of two citation databases: Scopus and Web of Science. Journal of the American Society for Information Science and Technology, 60(12), 2499–2508. doi: 10.1002/asi.21165

[20] Bar-Ilan J. (2008). Which h-index?—A comparison of WoS, Scopus and Google Scholar. Scientometrics, 74(2), 257–271. doi: 10.1007/s11192-008-0216-y

[21] Ortega, J. L. (2017). Reliability and accuracy of altmetric providers: a comparison among Altmetric, PlumX and Crossref Event Data. Retrieved from: https://osf.io/fuekb/

[22] National Information Standards Organization. (2016). Outputs of the NISO Alternative Assessment Metrics Project. Retrieved from http://www.niso.org/apps/group_public/download.php/17091/NISORP-25-2016OutputsoftheNISOAlternativeAssessmentProject.pdf

[23] Wass, J. (2017, 16 August). You do want to see how it’s made—seeing what goes into altmetrics. [Blog Post]. Retrieved from: http://altmetricsconference.com/you-do-want-to-see-how-its-made-seeing-what-goes-into-altmetrics/

[24] Wouters, P., & Costas, R. (2012). Users, narcissism and control—tracking the impact of scholarly publications in the 21st century. Utrecht: SURF foundation. Retrieved from http://www.surf.nl/nl/publicaties/Documents/Usersnarcissismandcontrol.pdf

[25] Fenner, M. (2013, 13 October). Challenges in automated DOI resolution [Blog Post]. Retrieved from: http://blog.martinfenner.org/2013/10/13/broken-dois/

[26] Peters I., Kraker P., Lex E, Gumpenberger C., and Gorraiz J.I. (2017). Zenodo in the Spotlight of Traditional and New Metrics. *Frontiers Research Metrics*. Anal. 2:13 doi: 10.3389/frma.2017.00013

[27] Robinson-García N., Torres-Salinas D., Zahedi Z., Costas R. (2014). New Data, New Possibilities: Exploring the Insides of Altmetric.Com. , 23(4), 359–366. doi: 10.3145/epi.2014.jul.03

[28] Batrinca B., & Treleaven P. C. (2015). Social media analytics: a survey of techniques, tools and platforms. AI & SOCIETY, 30(1), 89–116. doi: 10.1007/s00146-014-0549-4

[29] Lin J., Fenner M. (2013). Altmetrics in evolution: Defining and redefining the ontology of article-level metrics. Information Standards Quarterly, 25 (2) (2013), pp. 20–26 doi: 10.3789/isqv25no2.2013.04

[30] Carpenter, T. (2017, February 2). Plum Goes Orange: Elsevier Acquires Plum Analytics. [blog post]. The Scholarly Kitchen. Retrieved from: https://scholarlykitchen.sspnet.org/2017/02/02/plum-goes-orange-elsevier-acquires-plum-analytics/

[31] Haustein S., Bowman T. D., & Costas R. (2016). Interpreting Altmetrics: Viewing Acts on Social Media through the Lens of Citation and Social Theories In Sugimoto C. R. (Ed.), Theories of Informetrics and Scholarly Communication (pp. 372–406). Berlin, Boston: De Gruyter http://doi.org/10.1515/9783110308464-022

