# Supplementary material for: General discussion of data quality challenges in social media metrics: Extensive comparison of four major altmetric data aggregators
Source: PLoS One. 2018 May 17;13(5):e0197326. doi: 10.1371/journal.pone.0197326 (PMC5957428; doi:10.1371/journal.pone.0197326)
Supplement: S1 File — (PDF) [file pone.0197326.s001.pdf]

S2 Figs 1-2. Examples of records in Mendeley with incorrect PMIDs

S2 Fig 1. Examples of records in Mendeley with incorrect PMDI 25083704:

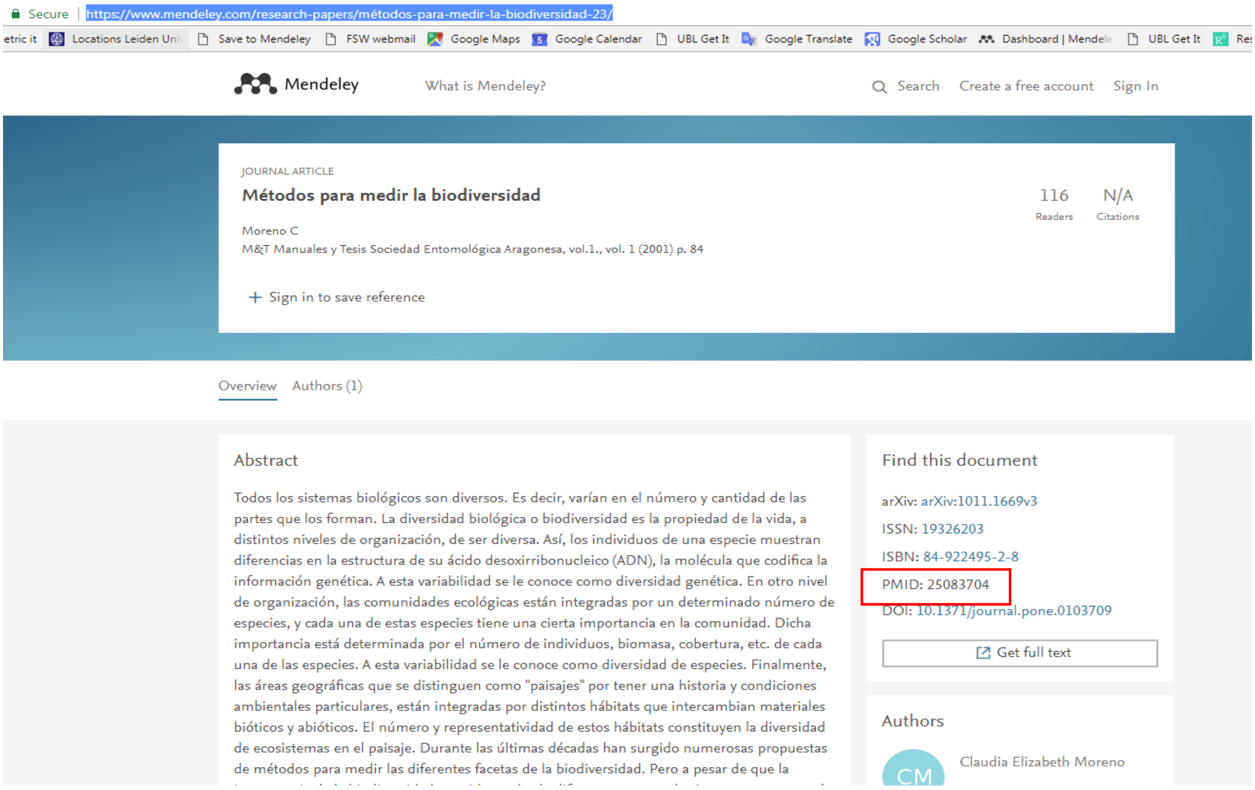

https://www.mendeley.com/research-papers/m%C3%A9todos-para-medir-la-biodiversidad-23/

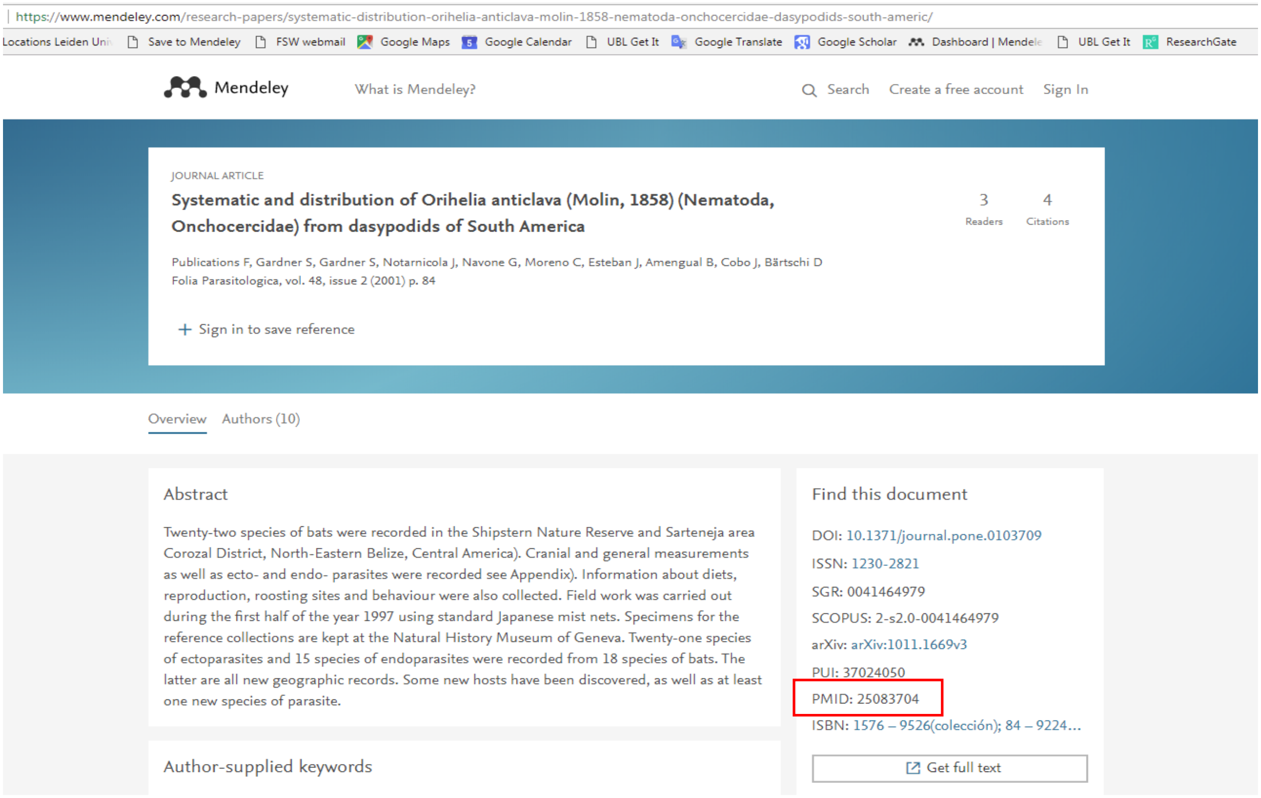

https://www.mendeley.com/research-papers/systematic-distribution-orihelia-anticlava-molin-1858-nematoda-onchocercidae-dasypodids-south-americ/

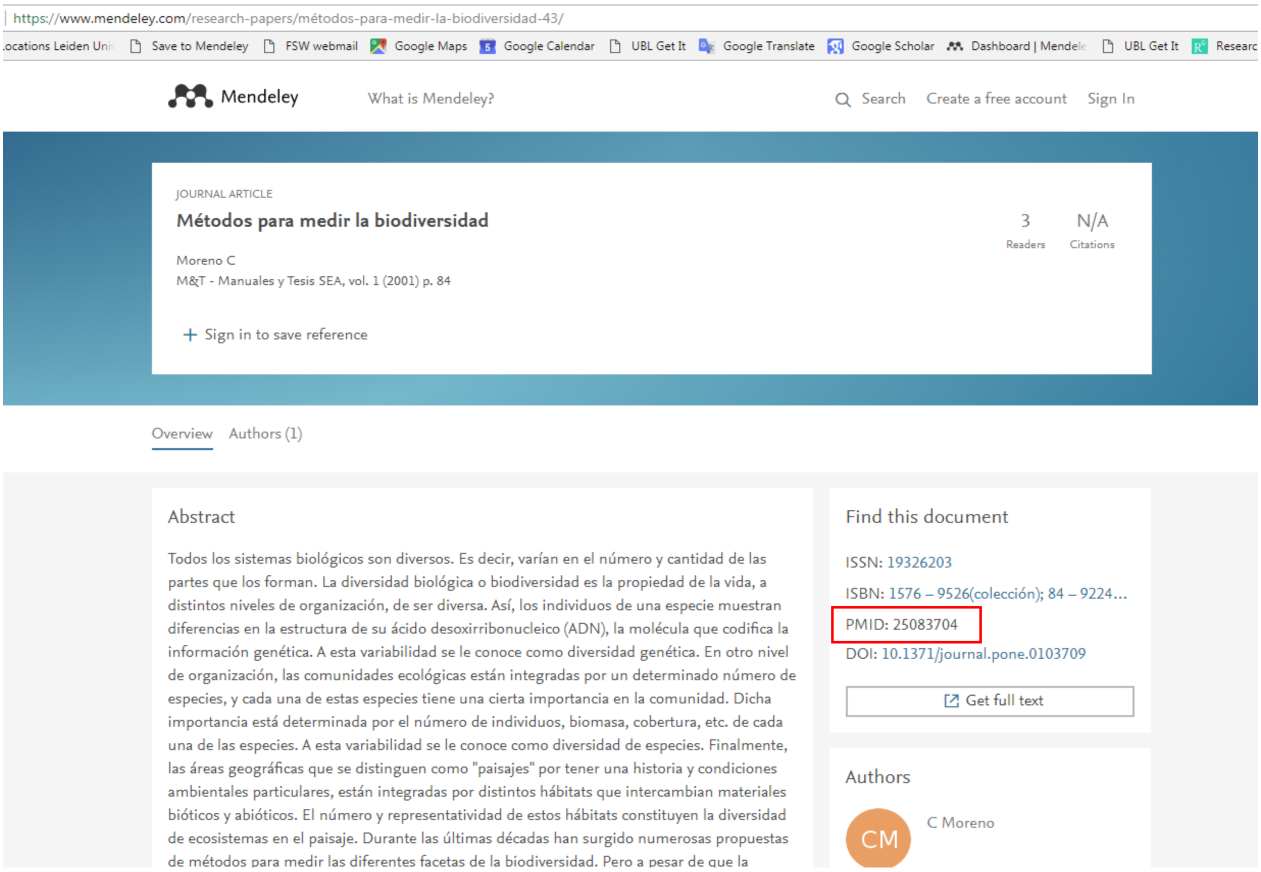

https://www.mendeley.com/research-papers/métodos-para-medir-la-biodiversidad-43/

.ocations Leiden Uni Save to Mendeley FSW webmail Google Maps Google Calendar UBL Get It Google Translate Google Scholar Dashboard | Mendele UBL Get It Researc

Mendeley What is Mendeley? Search Create a free account Sign In

JOURNAL ARTICLE

Métodos para medir la biodiversidad

3 Readers N/A Citations

Moreno C

M&T - Manuales y Tesis SEA, vol. 1 (2001) p. 84

+ Sign in to save reference

Overview Authors (1)

Abstract

Todos los sistemas biológicos son diversos. Es decir, varían en el número y cantidad de las partes que los forman. La diversidad biológica o biodiversidad es la propiedad de la vida, a distintos niveles de organización, de ser diversa. Así, los individuos de una especie muestran diferencias en la estructura de su ácido desoxirribonucleico (ADN), la molécula que codifica la información genética. A esta variabilidad se le conoce como diversidad genética. En otro nivel de organización, las comunidades ecológicas están integradas por un determinado número de especies, y cada una de estas especies tiene una cierta importancia en la comunidad. Dicha importancia está determinada por el número de individuos, biomasa, cobertura, etc. de cada una de las especies. A esta variabilidad se le conoce como diversidad de especies. Finalmente, las áreas geográficas que se distinguen como "paisajes" por tener una historia y condiciones ambientales particulares, están integradas por distintos hábitats que intercambian materiales bióticos y abióticos. El número y representatividad de estos hábitats constituyen la diversidad de ecosistemas en el paisaje. Durante las últimas décadas han surgido numerosas propuestas de métodos para medir las diferentes facetas de la biodiversidad. Pero a pesar de que la

Find this document

ISSN: 19326203

ISBN: 1576 – 9526(colección); 84 – 9224...

PMID: 25083704

DOI: 10.1371/journal.pone.0103709

Get full text

Authors

CM C Moreno

https://www.mendeley.com/research-papers/m%C3%A9todos-para-medir-la-biodiversidad-43/

S2 Fig 2. An example of a record in Mendeley with incorrect PMDI=25275510:

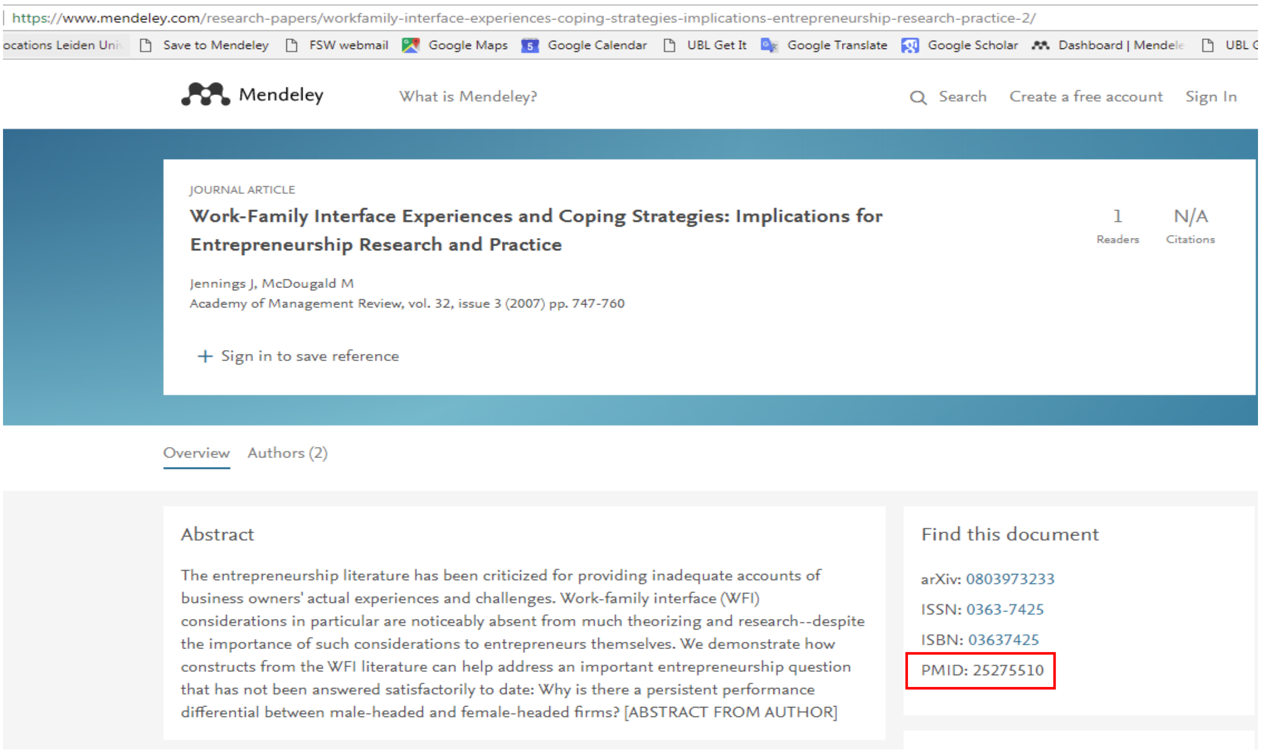

https://www.mendeley.com/research-papers/workfamily-interface-experiences-coping-strategies-implications-entrepreneurship-research-practice-2/

ocations Leiden Uni Save to Mendeley FSW webmail Google Maps Google Calendar UBL Get It Google Translate Google Scholar Dashboard | Mendele UBL G

Mendeley What is Mendeley? Search Create a free account Sign In

JOURNAL ARTICLE

Work-Family Interface Experiences and Coping Strategies: Implications for Entrepreneurship Research and Practice

1 Readers N/A Citations

Jennings J, McDougald M

Academy of Management Review, vol. 32, issue 3 (2007) pp. 747-760

+ Sign in to save reference

Overview Authors (2)

Abstract

The entrepreneurship literature has been criticized for providing inadequate accounts of business owners' actual experiences and challenges. Work-family interface (WFI) considerations in particular are noticeably absent from much theorizing and research--despite the importance of such considerations to entrepreneurs themselves. We demonstrate how constructs from the WFI literature can help address an important entrepreneurship question that has not been answered satisfactorily to date: Why is there a persistent performance differential between male-headed and female-headed firms? [ABSTRACT FROM AUTHOR]

Find this document

arXiv: 0803973233

ISSN: 0363-7425

ISBN: 03637425

PMID: 25275510

https://www.mendeley.com/research-papers/workfamily-interface-experiences-coping-strategies-implications-entrepreneurship-research-practice-2/
