# Supplementary material for: General discussion of data quality challenges in social media metrics: Extensive comparison of four major altmetric data aggregators
Source: PLoS One. 2018 May 17;13(5):e0197326. doi: 10.1371/journal.pone.0197326 (PMC5957428; doi:10.1371/journal.pone.0197326)
Supplement: S2 File — (PDF) [file pone.0197326.s002.pdf]

S1 texts 1-5. Extracts of methodological descriptions from Social Media platforms and Altmetric aggregators.

S1text 1

"The Twitter Search API is part of Twitter's REST API. It allows queries against the indices of recent or popular Tweets and behaves similarly to, but not exactly like the Search feature available in Twitter mobile or web clients, such as Twitter.com search. The Twitter Search API searches against a sampling of recent Tweets published in the past 7 days. Before getting involved, it's important to know that the Search API is focused on relevance and not completeness. This means that some Tweets and users may be missing from search results. If you want to match for completeness you should consider using a Streaming API instead[i] ."

S1text 2

"Altmetric.com tracks Twitter attention in real-time via an API. We collect tweets, retweets, and quoted tweets that contain a direct link to a scholarly output from a publisher we are tracking[ii]. It reports public tweets, retweets but not favorites or likes, that link directly to research outputs (protected tweets and any tweets prior to 2011 are not tracked)".

"We [Plum Analytics] license twitter data in PlumX directly through Twitter/GNIP. We have a filtered view of all tweets based upon the domain names of the links in the tweets. Our historic twitter data begins on January 1, 2011. We accommodate URL shorteners and have match and merge technology for combining tweets from multiple, separate URLs into a single view for a given artifact [object]. However, if the original artifact is published at a domain that we do not yet track, once identified and added by the Plum Analytics team, twitter mentions for that domain will only begin to be counted from the time the new domain is added[iii]".

"We [CrossRef ED] submit a set of filter rules to the Gnip Power Track service. This list is made up of DOI prefixes from the doi-prefix-list Artifact, DOI resolver domains, all domains in the domain-list Artifact (e.g. journals.iucr.org). The rules sent to Gnip Power Tack are manually updated. We aim to keep them in sync with the domain-list Artifact, but they may lag slightly. The Agent monitors all data sent back from the Power Track stream. This includes tweets that contain a DOI prefix, a hyperlinked DOI, Landing Page URL, or a link-shortened link to a DOI or Landing Page URL. The Gnip service automatically follows and extracts URLs from link-shortening services like bit.ly before the data is sent to us. This gives the Twitter source an advantage, as it removes opaque link-shortened links that we otherwise could not match. We then attempt to match all links to Registered Content Items. Publisher sites may block the Event Data Bot collecting Landing Pages[iv]".

"Lagotto searches the Twitter Search API by DOI and URL, e.g. The Search API will find shortened URLs with this query. The rate-limits for application-only authentication and search are 450 requests per 15 min or 1,800 requests per hour. Depending on the number of articles we might have to adjust how often we contact Twitter, the default settings are every 12 hours the first 7 days after publication, then daily for the first month, and then weekly[v]".

S1text 3

Plum Analytics:

"Online events about different versions of the same artifact [object](Publisher + Green Open Access + Preprint + Aggregated versions + A&I) are collected and aggregated based on algorithms that examine matching identifiers (such as DOI, ISBN, or URI) across versions. Usage, Capture, Social Media, and Mention metrics counts are summed across all versions of each artifacts. Our match and merge algorithms for combining and aggregating metrics from all the different online locations where it is published depend upon a knowledge base of how to cross-walk different identifiers (like going from a DOI to a PubMed ID). If there are errors in this crosswalk data, it is possible to "over-merge" a record. Similarly, if there is not enough data to automatically merge two preprints from two different services together, they may also need to be manually identified and merged by the PlumX staff [vi]".

S1text 4

"When a user deletes its account and all their documents, the readership of that document doesn't change, until the batch clustering process is re-run and the new number of metadata records is generated. The same applies when a user deletes a record from its library. In summary, the count of records can increase nearly instantaneously, but only decreases periodically [vii]".

S1text 5

"These identifiers also help us [altmetric.com] to recognize different versions of the same research output. For example, a journal article might be originally made available on a publisher platform and given a DOI, and then later hosted on PubMed or an institutional repository and given another unique identifier there. Our system cross-checks these to match them together, ensuring that the details page always displays a collated record of attention for all versions of the research item[viii]".

---

[i] https://developer.twitter.com/en/docs/tweets/rules-and-filtering/guides/how-to-build-a-query

[ii] https://help.altmetric.com/support/solutions/articles/6000157183-how-does-altmetric-track-twitter-

[iii] https://plumanalytics.com/niso-altmetrics-working-group-on-data-quality/

[iv] https://www.eventdata.crossref.org/guide/sources/twitter/

[v] http://www.lagotto.io/docs/twitter_search/

[vi] https://plumanalytics.com/niso-altmetrics-working-group-on-data-quality/

[vii] www.niso.org/apps/group_public/view_comment.php?comment_id=632;
www.niso.org/apps/group_public/view_comment.php?comment_id=610

[viii] https://www.altmetric.com/about-our-data/how-it-works/
