# Supplementary material for: General discussion of data quality challenges in social media metrics: Extensive comparison of four major altmetric data aggregators
Source: PLoS One. 2018 May 17;13(5):e0197326. doi: 10.1371/journal.pone.0197326 (PMC5957428; doi:10.1371/journal.pone.0197326)
Supplement: S3 File — (PDF) [file pone.0197326.s003.pdf]

S3 text. Excerpt of the JSON file from CrossRef ED recording 315 Wikipedia mentions for an object with DOI: 10.1371.journal.pone.0105090):

{"status":"ok","message-type":"event-list","message":{"next-cursor":"0d2917ad-89d6-4bd5-a57b-36af574e8510","total-results":315,"items-per-page":1000,"events":[{"license":"https:\/\/creativecommons.org\/publicdomain\/zero\/1.0\/","obj_id":"https:\/\/doi.org\/10.1371\/journal.pone.0105090","source_token":"36c35e23-8757-4a9d-aacf-345e9b7eb50d","occurred_at":"2017-04-18T14:49:16Z","subj_id":"https:\/\/mk.wikipedia.org\/w\/index.php?title=%D0%9C%D0%B0%D0%BA%D0%B5%D0%B4%D0%BE%D0%BD%D1%86%D0%B8&oldid=3586676","id":"82ed5180-b2ee-486a-b495-aa2883c1b1be","evidence_record":"https:\/\/evidence.eventdata.crossref.org\/evidence\/20170418-wikipedia-4a99bd66-e33f-49c5-a52a-0b3690c3e5a0","terms":"https:\/\/doi.org\/10.13003\/CED-terms-of-use","action":"add","subj":{"pid":"https:\/\/mk.wikipedia.org\/w\/index.php?title=%D0%9C%D0%B0%D0%BA%D0%B5%D0%B4%D0%BE%D0%BD%D1%86%D0%B8&oldid=3586676","url":"https:\/\/mk.wikipedia.org\/wiki\/%D0%9C%D0%B0%D0%BA%D0%B5%D0%B4%D0%BE%D0%BD%D1%86%D0%B8","title":"\u041c\u0430\u043a\u0435\u0434\u043e\u043d\u0446\u0438","api-url":"https:\/\/mk.wikipedia.org\/api\/rest_v1\/page\/html\/%D0%9C%D0%B0%D0%BA%D0%B5%D0%B4%D0%BE%D0%BD%D1%86%D0%B8\/3586676"},"source_id":"wikipedia","obj":{"pid":"https:\/\/doi.org\/10.1371\/journal.pone.0105090","url":"https:\/\/doi.org\/10.1371\/journal.pone.0105090"},"timestamp":"2017-04-18T14:50:20Z","relation_type_id":"references"}, ….
